# Comparison of extraction methods for per- and polyfluoroalkyl substances (PFAS) in human serum and placenta samples—insights into extractable organic fluorine (EOF)

**DOI:** 10.1007/s00216-020-03041-5

**Published:** 2020-11-19

**Authors:** Andreas-Marius Kaiser, Rudolf Aro, Anna Kärrman, Stefan Weiss, Christina Hartmann, Maria Uhl, Martin Forsthuber, Claudia Gundacker, Leo W. Y. Yeung

**Affiliations:** 1 Environment Agency Austria, Spittelauer Lände 5, 1090 Vienna, Austria; 2 Institute of Medical Genetics, Center for Pathobiochemistry and Genetics, Medical University of Vienna, 1090 Vienna, Austria; 3 Man-Technology-Environment Research Centre (MTM), Örebro University, 701 82 Örebro, Sweden; 4 Department of Environmental Health, Center for Public Health, Medical University of Vienna, 1090 Vienna, Austria

**Keywords:** Human serum, Human placental tissue, Perfluoroalkyl substances, Extractable organic fluorine

## Abstract

Since the detection of per- and polyfluoroalkyl substances (PFAS) in humans and different environmental media in the last two decades, this substance group has attracted a lot of attention as well as increasing concerns. The fluorine mass balance approach, by comparing the levels of targeted PFAS after conversion to fluorine equivalents with those of extractable organic fluorine (EOF), showed the presence of unidentified organofluorine in different environmental samples. Out of the thousands of PFAS in existence, only a very small fraction is included in routine analysis. In recent years, liquid chromatography coupled with tandem-mass spectrometry (LC-MS/MS) has demonstrated the ability to analytically cover a wide spectrum of PFAS. In contrast, conventional extraction methods developed 10 to 15 years ago were only evaluated for a limited number of PFAS. The aim of the present study was to evaluate the advantages and disadvantages of three different extraction methods, adapted from the literatures without further optimization (ion-pair liquid-liquid extraction, solid-phase extraction (SPE), using hydrophilic-lipophilic (HLB) or weak anion exchange (WAX) sorbents), for human biomonitoring of 61 PFAS in serum and placental tissue samples. In addition, levels of EOF were compared among these extraction methods via spiked samples. Results showed that performance, in terms of recovery, differed between the extraction methods for different PFAS; different extraction methods resulted in different EOF concentrations indicating that the choice of extraction method is important for target PFAS and EOF analysis. Results of maternal serum samples, analyzed in two different laboratories using two different extraction methods, showed an accordance of 107.6% (± 21.3); the detected perfluoroalkyl acids (PFAAs) in maternal and cord serum samples were in the range of 0.076 to 2.9 ng/mL.

Graphical abstract

**Supplementary Information:**

The online version contains supplementary material available at 10.1007/s00216-020-03041-5.

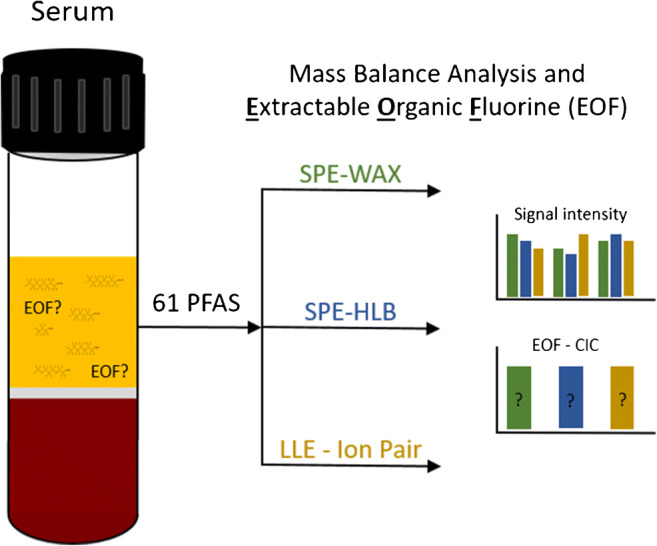

## Introduction

Per- and polyfluoroalkyl substances (PFAS) are a group of (mostly) man-made compounds, produced since the 1950s, and consist of carbon chains with fluorine atoms and at least one functional group; they have been used for a variety of industrial and consumer applications, such as cosmetics, clothing, and food packaging materials [1, 2]. Due to the strong C–F bond, PFAS are resistant to thermal, chemical, and biological degradation [3], making them prone to bioaccumulation and long-range environmental transport [4]. A large number of studies have reported the occurrence of PFAS in different environmental media [5, 6] and humans [4, 7–11], as well as their suspected toxic properties, since the turn of the twenty-first century. Global contamination of PFAS is considered to be totally anthropogenic [12] since the occurrence of natural organofluorine compounds is exceedingly rare. A handful of organofluorine compounds are produced in nature by very few tropical plants [13], in volcanic gases and drill wells, where they contain one to four fluorine atoms [14].

Studies on animals as well as epidemiological studies have identified various adverse health effects for several PFAS, such as hepatotoxicity, developmental toxicity, immunotoxicity, endocrine-disrupting effects, reprotoxicity, and carcinogenicity [6, 15–18]. The primary manufacturer phased out the production of perfluorooctane sulfonic acid (PFOS) and PFOS-based products since 2000 and agreed to phase out perfluorooctanoic acid (PFOA) as well as any products, that will degrade into PFOA or related higher homologues (i.e., more than seven fluorinated carbons), by 2015 [19]. Meanwhile, PFOS and PFOA, as well as their salts and related substances, were added to the list of persistent organic pollutants (POPs), under the Stockholm Convention in 2009 and 2019, respectively [20]. Due to global regulations, the production of PFOA, PFOS, and related compounds shifted from the USA and Europe to China, where the production continued in large quantities [21, 22]. Moreover, shorter chain-length PFAS (C4–C6) as well as other new compounds, such as polyfluoroalkyl ether acids, are increasingly used as dominant alternative compounds [23, 24]. Nowadays, more than 4700 different PFAS are registered on the global market [25]. Although human biomonitoring studies have reported a decline in human exposure to PFOA and PFOS during past years [26, 27], it is still of concern due to inconsistent trends of other long-chain PFAAs, such as perfluorononanoic acid (PFNA), perfluorodecanoic acid (PFDA), and perfluoroundecanoic acid (PFUnDA), as reported in humans in some studies [28, 29], while other studies reported declining trends [30, 31]. However, these substances are also a subject of regulatory activities in the European Union [32].

PFOA alternatives, such as polyfluoroalkyl ether carboxylic acids (e.g., dodecalfluoro-3H-4,8-dioxanonanoate (ADONA)), were detected in human serum [23] and perfluoroalkyl ether carboxylic acids (e.g., hexafluoropropylene oxide dimer acid (HFPO-DA, also known as GenX), as well as 6:2 chlorinated polyfluoroalkyl ether sulfonates (6:2 Cl-PFESA, also known as F53B)) were found in environmental samples in Europe and China [24, 33]. Additionally, other new and mostly unknown substances are continuously identified in water bodies [34]. Thus, humans might be exposed to various new and/or unknown PFAS, which makes monitoring of these compounds in the environment and biota necessary.

During the past years, liquid chromatography coupled with mass spectrometry (LC-MS) instrumentation has improved; new column materials became available to diminish interferences between co-extracts (e.g., taurodeoxycholic acid) and targeted compounds (e.g., PFOS). Furthermore, more mass-labeled standards for quantification became available as well, which led to production of more reliable results. It is impractical to measure all possible individual PFAS in one sample, as new PFAS are continuously introduced to the global market. The measurement of extractable organic fluorine (EOF), first presented by Miyake and co-workers, is a promising concept to estimate the total PFAS content in a sample [35]. In short, in fluorine mass balance analysis, the measured EOF (via combustion ion chromatography (CIC)) and targeted PFAS (via LC-MS/MS) levels, after conversion into fluorine equivalents, are compared; the difference between EOF and targeted PFAS indicates the amount of unidentified organic fluorine.

EOF has been detected in human blood using the ion-pair liquid-liquid extraction method [26] and protein precipitation with acetonitrile followed by graphitized non-porous carbon cleanup [30], but not yet by using solid-phase extraction (SPE) procedures. Since different sample pretreatments and extraction methods will result in different fractions of EOF, this investigation compared the amounts of EOF in spiked serum and human placental tissue samples using various extraction methods, to identify the type of organic fluorine the EOF is representing.

The objectives of this study were (i) to compare the extraction efficiencies of different extraction procedures (adapted from literatures without further optimization), which included an ion-pair liquid-liquid extraction method and SPE with weak anion exchange (WAX) or hydrophilic-lipophilic balance (HLB) sorbents, for the analytical determination of 61 target PFAS as well as EOF using bovine serum and human placental tissue samples, and (ii) to compare the results of PFAS concentrations identified in eight maternal serum samples using two different extraction methods (i.e., ion-pair and SPE-HLB) and two different instruments (i.e., a triple quadrupole instrument and a hybrid triple quadrupole/linear ion trap). This paper discusses advantages and disadvantages of the different extraction methods for EOF and PFAS analyses. There is an interest to evaluate human exposure to polyfluoroalkyl phosphate esters (PAPs), and therefore, eight corresponding maternal and cord serum samples were analyzed with the extraction method showing the best performance for PAPs.

## Materials and methods

### Sample collection

The plasma and placental tissue samples used for the present study were collected in the frame of an Austrian mother-child pair study, between 2017 and 2019 at the Vienna General Hospital (AKH Vienna). The participating mothers were aged 18–45 years and had a healthy singleton pregnancy. Maternal blood samples were collected a few days to a few hours before delivery. The cord blood samples and placental tissue samples were collected after the umbilical cord was tied. The study was approved by the Ethics Committee of the Medical University of Vienna and the Vienna General Hospital (EK No. 1035/2015). Written informed consent was obtained from all participants by medical personnel. The blood samples were centrifuged within the first hour to obtain serum and stored at − 20 °C until the analyses. In total, eight maternal serum samples and eight cord serum samples of corresponding mother-child pairs were included in the present investigation. One additional placental tissue sample was used, although the corresponding serum samples were not available.

### Chemical analysis

The analytical work had been performed in two different research laboratories (Environment Agency Austria and MTM Research Centre) as described below**.**

#### Environment Agency Austria

For the serum and placental tissue samples, 31 PFAS were analyzed, including substances from different classes: perfluorocarboxylic acids (PFCAs: C4–C14), perfluorosulfonic acids (PFSAs: C4–C10), perfluorooctane sulfonamides (perfluoro-n-octane sulfonamide (FOSA), *N-*ethyl-perfluoro-n-octane sulfonamide (EtFOSA)), *N-*ethyl-perfluoro-n-octane sulfonamido acetic acid (EtFOSAA), *N-*ethyl-perfluoro-n-octane sulfonamido ethanol (EtFOSE), fluorotelomer sulfonates (FTSAs: 4:2, 6:2, and 8:2), polyfluoroalkyl phosphate diesters (diPAPs: 6:2/6:2, 6:2/8:2, 8:2/8:2), polyfluorinated ether acids (ADONA and GenX), and 6:2 Cl-PFESA (F-53B)). Detailed information on the substances and mass-labeled internal standards is given in the Electronic Supplementary Material (ESM). All used standards were purchased from Wellington Laboratories (Ontario, Canada) with a purity ≥ 98%. Adult bovine serum was purchased from Sigma-Aldrich® (St. Louis, MO, USA). Chemicals used are provided in the ESM.

#### Sample preparation of serum samples

**Fig. 1:**
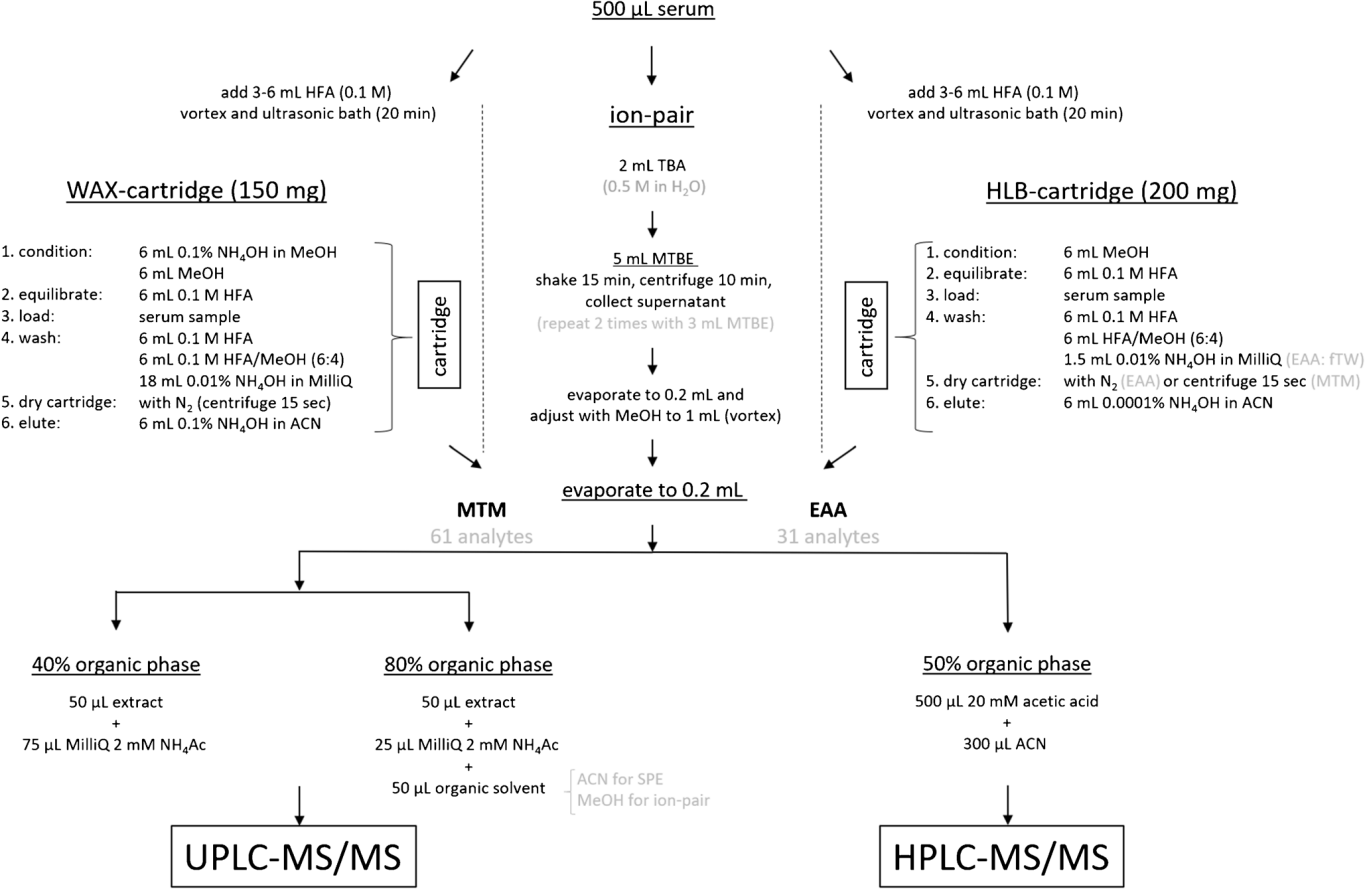
Methodological procedures for the SPE-WAX, ion-pair, and SPE-HLB methods applied at the MTM Research Centre and the Environment Agency Austria (EAA). At the EAA, only the SPE-HLB was applied whereas all three methods were tested at the MTM

The solid-phase extraction using hydrophilic-lipophilic balance sorbent (SPE-HLB) was adapted from the work of Kuklenyik and co-workers [36], with modifications. In short, 500 μL of serum sample was transferred into a polypropylene (PP) tube and spiked with 10 ng of mass-labeled standards (10 ng of each compound, see ESM Table S1). The samples were mixed (vortex) before and after adding 3 mL of 0.1 M formic acid (HFA) solution in filtered tap water, and ultrasonicated for 20 min, followed by SPE-HLB. The schematics and details of the SPE-HLB procedures are provided in Fig. 1 and the ESM, respectively. The final extract volume was reduced to 500 μL under a mild nitrogen flow at 40 °C and was adjusted to 1 mL with 20 mM acetic acid (HAC) solution in filtered tap water for instrumental analysis.

#### Sample preparation of placental tissue samples (placenta I method)

The preparation of placental tissue was adapted based on the method developed by Martin and co-workers [37]. The placental tissue samples were cut on Petri dishes pre-cleaned with methanol until the tissue had a homogeneous and almost gelatinous texture. The samples were spiked with 10 ng of the mass-labeled standards and a PFAS standard mix (5 ng of each substance), as given in the ESM (Table S1). Further, the samples were freeze-dried for 48–60 h and stored at − 20 °C, if not immediately analyzed. Two hundred microliters of 0.5 M formic acid in filtered tap water and 5 mL of acetonitrile were added to 2.5 g of the freeze-dried placental tissue. Then, the tissue was loosened in a PP tube, with a Pasteur pipette, followed by mixing by vortexing and ultrasonication for 20 min. After ultrasonication, the sample was centrifuged for 15 min at 4700 rpm. 3.4 mL of the supernatant was transferred into the PP tube, containing 100 mg graphitized non-porous carbon (EnviCarb), and was shaken for 1 min. After that, the sample was centrifuged for an additional 15 min and 3.0 mL of the supernatant was transferred into another PP tube. The final extract volume was reduced to 500 μL, under a mild nitrogen flow at 40 °C, and was then adjusted to 1 mL with 20 mM HAC solution in filtered tap water. The samples were transferred into PP vials for instrumental analysis.

#### Instrumental analysis of targeted PFAS

The targeted analysis of PFAS in the serum and placental tissue samples was performed by high-performance liquid chromatography coupled with tandem-mass spectrometry (HPLC-MS/MS). This analytical system was composed of an Agilent Technologies 1290 Infinity Series (Agilent Technologies, Santa Clara, CA, USA) HPLC and a SCIEX 4000 QTRAP mass spectrometer (AB Sciex Technologies, Framingham, MA, USA) in electrospray ionization (ESI) negative mode. The analytical column was a Luna 5 μm C18(2), 100 × 2 mm (Phenomenex, CA, USA). The eluents were methanol (mobile phase B) and LC-MS grade water, containing 10 mM ammonium acetate (mobile phase A). Details of the LC program are provided in the ESM.

#### MTM Research Centre

Three different extraction methods (SPE-WAX, SPE-HLB, and ion-pair) for the serum samples and one for the placental tissue samples were evaluated for 61 PFAS. The 61 target PFAS included PFCAs (C4–C14, C16, C18), PFSAs (C4–C10, C12), N- and Me-perfluorobutane sulfonamides (FBSAs), perfluorohexane sulfonamides (FHxSAs: N- and Me-), FOSAs (N-, Me-, and Et-), FOSAAs (Et- and Me-), FOSEs (Me- and Et-), FTSAs (4:2, 6:2, and 8:2), fluorotelomer saturated/unsaturated acids (FTCAs: 3:3, 5:3, and 7:3 and FTUCAs: 6:2, 8:2, and 10:2), 6:2 polyfluoroalkyl phosphoric acid monoester (monoPAPs: 6:2, 8:2, 10:2), polyfluoroalkyl phosphate diesters (diPAPs: 6:2/6:2, 6:2/8:2, 8:2/8:2, 10:2/10:2), (bis-)2-N-ethylperfluorooctane-1-sulfonamido-ethyl-phospate (diSAmPAP), polyfluorinated ether acids (ADONA, HFPO-DA (GenX)), and chlorinated polyfluorinated ether sulfonates (6:2 and 8:2)), perfluorinated phosphonic acids (PFPAs: C6, C8, C10), perfluorinated phosphinic acids (6:6, 6:8 and 8:8), and perfluoro-4-ethylcyclohexane (PFECHS). The analytical standards used were purchased from Wellington Laboratories (Ontario, Canada), with a purity ≥ 96%, and from Apollo Scientific Ltd. perfluorobutane sulfonamide (FBSA), with a purity ≥ 97%.

#### Sample preparation for serum samples

For the analysis of the maternal and cord serum samples, three different extraction procedures were applied and compared.

##### SPE-HLB

This extraction method followed the SPE-HLB method discussed above with some exceptions: the cartridges were centrifuged for 15 s at 6000 rpm to remove remaining residual water instead of drying under a nitrogen flow. The schematics of the SPE-HLB procedures are provided in Fig. 1.

##### SPE-WAX

An SPE was carried out with weak anion exchange sorbent (SPE-WAX), based on methods published by Kuklenyik and co-workers [36] and Miyake and co-workers [35], with modifications. Five hundred microliters of the sample was transferred to PP tubes and spiked with internal standards; they were vortexed before and after the addition of 6 mL of 0.1 M HFA solution in Milli-Q water and subsequently ultrasonicated for 15 min. The schematics and details of the SPE-WAX procedures are provided in Fig. 1 and the ESM. The final extract volume was reduced to 200 μL and was split according to Fig. 1 for instrumental analysis.

##### An ion-pair method

A modified ion-pair sample preparation method, published by Hansen and co-workers [38], was used. Five hundred microliters of the serum sample was spiked with 2 ng (each compound) of the internal standard mix II (see ESM). The schematics and details of the ion-pair procedures are provided in Fig. 1 and the ESM. The final extract volume was reduced to 200 μL and split, according to Fig. 1, for instrumental analysis.

#### Sample preparation of the placental tissue samples (placenta II method)

The sample treatment of the placental tissue was similar to the description above, with the following modifications: 3 g placental tissue instead of 2.5 g and 8 mL of ACN instead of 5 mL were used. The cleanup was performed using a 250-mg EnviCarb cartridge. First, the EnviCarb cartridge was conditioned with 2 mL ACN; then, the sample was applied onto the cartridge and collected. Next, 2 mL ACN was applied. The final extract volume was reduced to 200 μL and split, according to Fig. 1, for instrumental analysis.

#### Instrumental analysis of targeted PFAS

The chemical analysis of targeted PFAS was performed by an ultra-performance liquid chromatography (UPLC) system from Waters (Acquity UPLC®, Waters Corporation, Milford, MA, USA), coupled to either a Xevo TQ-S or a Xevo TQ-S-micro mass spectrometer in ESI negative mode. The stationary phase was an ACQUITY UPLC® BEH C18 1.7 μm, 2.1 × 100 mm column (Waters Corporation, Milford, MA, USA), and the eluents for the mobile phases were a 70:30 mixture of Milli-Q water and methanol (mobile phase A) and methanol (mobile phase B), both containing 2 mmol/L ammonium acetate and 5 mmol/L n-methylpiperidine (only in Xevo TQ-S). Details of the LC program are provided in the ESM.

#### Sample preparation for extractable organic fluorine analysis

Extractable organic fluorine (EOF) was determined in spiked bovine serum, after different extraction procedures (SPE-WAX, SPE-HLB, and ion-pair extraction method) were applied, as well as in placental tissue samples, using the placenta II method, as described above. After extraction, 60 μL of the final extract (see Fig. 1) was mixed with 60 μL of the particular organic solvent (MeOH for the ion-pair extraction method, and ACN for the SPE-WAX, SPE-HLB, and placenta II method). ESM Fig. S3 shows the elements of the total fluorine content in one sample and the resulting EOF fraction after the extraction procedure.

#### Instrumental analysis of EOF

A combustion ion chromatography (CIC) was used to analyze the EOF content in the samples. The instrument consisted of a combustion module (Analytik Jena, Germany), a 920 Absorber Module, and a 930 Compact IC Flex ion chromatograph (Metrohm, Switzerland). The ion exchange column was a Metrosep A Supp 5 – 150/4.0 (Metrohm, Switzerland), and the eluent was an isocratic elution using a carbonate buffer (64 mmol/L sodium carbonate and 20 mmol/L sodium bicarbonate, Sigma-Aldrich®, St. Louis, USA). Details of the instrumental analysis are provided in the ESM.

### Comparisons of extraction methods

For the evaluation of the performance of the different extraction methods, the procedure blanks, the ion signal effects (ion suppression/enhancement), and the recoveries were used. The ion signal effects were evaluated by comparison of the peak area of recovery of the mass-labeled standards of PFAS (2 ng) spiked bovine serum after extraction and the recovery of the mass-labeled standards of PFAS in the solvent (2 ng) multiplied by 100%, whereas > 100% indicates ionization enhancement, and < 100% indicates ionization suppression. The precisions were assessed via the standard deviation among replicates. The recoveries were evaluated in two different ways: (i) *recovery based on mass-labeled standard* refers to the peak area of the internal mass-labeled standard (IS), divided by the corresponding recovery mass-labeled standard (RS) in a sample multiplied by 100%, whereas (ii) *recovery based on peak area* refers to the peak area of a native compound in a spiked sample divided by the peak area of the same native compound in a solvent (e.g., organic phase water mixture) multiplied by 100%.

Evaluation of the extraction methods was extended to EOF analysis by measuring EOF levels in spiked extracts. The evaluation criteria included EOF concentrations measured as well as the standard deviations in the replicates.

### Quality assurance and control measures

In PFAS analysis, several quality assurance and control (QA/QC) measures were included, comparing extraction blanks and the measurement of quality control (QC) samples. Three QC samples and two extraction blanks were measured in each batch. The extraction blank was Milli-Q water and the QC sample bovine serum spiked with the compounds listed in ESM Table S3 (1 ng each compound). Furthermore, the cartridges from the SPE-HLB QCs were separately eluted again, using 0.1% and 1% NH_4_OH in 4 mL ACN to evaluate potential recovery differences, with the focus on long-chain compounds. The same was done with three QCs from the SPE-WAX method with 1% NH_4_OH in 4 mL ACN. The limit of quantification (LOQ) was determined as a signal-to-noise ratio of ≥ 10, considering the specific recoveries. In addition, it was verified that all LOQs were at least 5 times higher than the blank [39]. The LOQs were furthermore adjusted for sample volumes. For the reported limits of detection (LODs), the evaluated LOQs were divided by two (see ESM Table S3). The calibration curve included ten concentrations ranging between 0.005 and 30 ng/mL. Multiple reaction monitoring (MRM) was used and at least two transitions were monitored for all analytes, except for PFBA, PFPeA, and EtFOSE where one transition was monitored. Results were only reported if both transitions were detected and their ratio was within 50% of that observed in a standard. In the present investigation, compounds that showed a recovery of ≥ 30% were considered acceptable. Information on the elimination of inorganic fluorine is provided in the ESM (Table S9).

For the analysis of EOF, multiple measurements of combustion blanks were started until the combustion blanks showed low variability (below 5% relative standard deviation (RSD)) over the last three combustion blanks, due to the CIC system contained background fluoride contamination. All measurements of samples were first subtracted from the combustion blanks between samples before quantification, using an external calibration curve. The calibration curve which included five concentrations ranging between 50 ng/mL fluorine (F) and 1000 ng/mL was constructed with a PFOS standard.

### Statistical analysis

The statistical analysis was performed with R version 3.5.2. The Shapiro-Wilk normality test was used to test if the distributions were significantly different from a normal distribution. The Wilcoxon signed-rank test was used to assess statistically significant differences in PFAS concentrations in serum samples measured with the different analytical methods. Statistically significant correlations were investigated, using Pearson’s correlation test in case the results showed normal distribution, and using Spearman’s correlation test in case the results were not normally distributed (results shown in the ESM). For the statistical analysis, the measured substance concentrations <LOD were set at 0, and concentrations <LOQ and >LOD were set at LOQ/√2.

## Results and discussion

### Performance of different extraction methods

#### Spiked bovine serum samples

##### Blank level

No detectable levels (< 0.020–< 2.0 ng/mL) of target PFAS were found in any of the procedure blanks of the extraction methods investigated (see ESM Table S5).

##### Signal effect in spiked bovine serum

Effects on the ionization (enhanced or suppressed ion signal) were evaluated on the nine surrogate mass-labeled standards, using the three extraction methods, and are summarized in Table 1. Both ionization enhancement and suppression were observed on these nine surrogate standards, depending on the respective extraction method. Ionization suppression of up to 14.1% was observed for some compounds extracted by the ion-pair method, whereas some compounds showed enhanced signals using SPE methods up to 23.3% for WAX and up to 34.5% for HLB. The causes of ionization suppression have been reviewed by Furey and co-workers [40] and, as discussed in a previous study [41], the ion-pair method is known to co-extract a lot of matrix components which might lead to co-elution of interfering substances resulting in suppressed ionization of target analytes. On the other hand, SPE methods were shown to have minimal ionization suppression and slight ionization enhancement [40], which is similar to the results observed in the present investigation. It should be kept in mind that the ion signal effect is also affected by the ion source design (e.g., linear, orthogonal, or Z-spray) and the chromatographic separation conditions. A further cleanup step is needed for the ion-pair method, especially for trace level analysis. The use of suitable or corresponding mass-labeled standards is needed to correct the ion signal effect during instrumental analysis.

**Table 1:** The recoveries and signal effects in %, with the respective standard deviation in brackets—*n* = 3 for all mass-labeled substances and methods, except for PFUnDA from the ion-pair method, which was *n* = 2

Compound	Ion-pair (*n* = 3)			SPE-WAX (*n* = 3)			SPE-HLB (*n* = 3)		
	Recovery (%)	Signal effects (%)	conc. (ng/mL)	Recovery (%)	Signal effects (%)	conc. (ng/mL)	Recovery (%)	Signal effects (%)	conc. (ng/mL)
PFBA	54.4 (± 4)	− 3.2 (± 14)	2.0 (± 0.20)	83.9 (± 5)	2.4 (± 6)	2.0 (± 0.14)	53.3 (± 11)	34.5 (± 24)	2.0 (± 0.69)
PFPeA	42.0 (± 2)	− 2.0 (± 12)	1.6 (± 0.052)	65.6 (± 3)	8.1 (± 6)	2.0 (± 0.11)	51.0 (± 9)	27.2 (± 18)	2.0 (± 0.55)
PFHxA	47.0 (± 2)	− 14.1 (± 11)	1.5 (± 0.027)	68.8 (± 5)	11.0 (± 5)	1.9 (± 0.17)	59.2 (± 10)	7.5 (± 9)	2.0 (± 0.36)
PFOA	47.6 (± 2)	− 9.7 (± 10)	2.0 (± 0.056)	68.8 (± 3)	17.0 (± 7)	1.9 (± 0.14)	57.8 (± 9)	11.6 (± 9)	2.0 (± 0.37)
PFNA	46.1 (± 1)	− 6.4 (± 7)	2.0 (± 0.000)	71.3 (± 2)	11.2 (± 6)	2.0 (± 0.080)	59.4 (± 10)	3.0 (± 9)	2.0 (± 0.37)
PFDA	41.6 (± 2)	14.2 (± 15)	1.9 (± 0.062)	70.7 (± 2)	23.3 (± 7)	2.0 (± 0.095)	58.4 (± 10)	33.4 (± 10)	2.0 (± 0.36)
PFUnDA	39.5	13	2.0	67.6	22.7	2.0	56.4	24.7	2.0
PFHxS	72.8 (± 1)	− 12.2 (± 11)	1.9 (± 0.075)	72.4 (± 2)	15.1 (± 8)	1.9 (± 0.050)	62.2 (± 5)	27.5 (± 12)	1.9 (± 0.14)
PFOS	68.4 (± 6)	4.8 (± 15)	2.4 (± 0.24)	80.6 (± 1)	16.3 (± 8)	2.0 (± 0.091)	70.5 (± 6)	34.2 (± 15)	2.0 (± 0.20)

##### Recovery

**Fig. 2:**
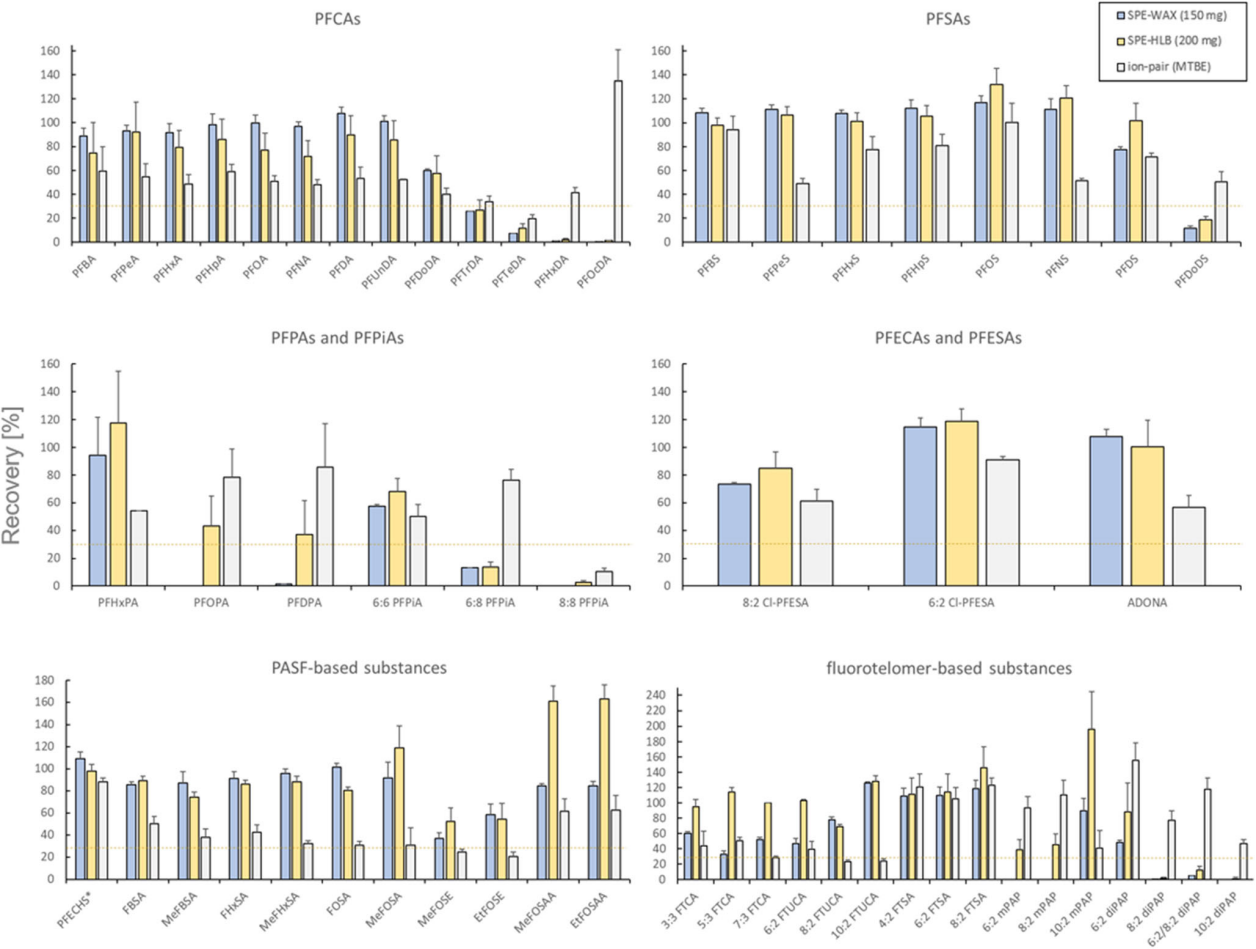
The recoveries in % are based on the comparison of the sample peak area with the calibration standard peak area. For all substances and methods *n* = 3, except for PFUnDA, PFOcDA, PFNS, PFECHS, MeFOSE, EtFOSE, PFHxPA, 6:8 PFPiA, 8:8 PFPiA, and 6:2 Cl-PFESA for the ion-pair method which were *n* = 2

Recoveries from the three extraction methods were evaluated in two different ways, as explained above: (i) the nine surrogate mass-labeled standards are summarized in Table 1 and (ii) the 61 native substances are summarized in Fig. 2.

In brief, the SPE-WAX showed the best recoveries for the nine surrogate mass-labeled standards with an average of 75% (range: 65–84%). The ion-pair method resulted in lower average recoveries of 51% (range: 39%–73%). While the ion-pair method had comparable recoveries with SPE-WAX and SPE-HLB for PFSAs, lower recoveries of PFCAs were noted, especially for short-chain PFCAs. The average recoveries were comparable between SPE-HLB (59%) and ion-pair (51%). However, the recoveries of the SPE-HLB method were slightly higher for the PFCA, whereas the ion-pair method showed similar recoveries for PFSAs. The ion-pair method was shown to have comparable recoveries with SPE-WAX for PFSAs but not for PFCAs, with lower average recoveries at 24%. This has been shown especially for PFBA, for which the recovery was nearly 30% lower, using the ion-pair method compared to the SPE-WAX method. It is reasonable to get these results as SPE-WAX has been shown to be able to capture ultra-short to long-chain PFAAs in water samples, due to the ion exchange capacity [42, 43], whereas SPE-HLB has been shown to result in slightly lower recoveries of short-chain compounds [42, 43]. The recovery of the ion-pair method depends on the formation of a stable ion-pair between the target analytes and the ion-pair reagent. Some short-chain PFAAs might not be able to form a stable ion-pair because of their hydrophilic nature and some long-chain PFAAs might preferably interact with matrix particulates, which resulted in lower recoveries. The use of suitable or corresponding mass-labeled standards is needed to correct recovery losses during the extraction.

The recoveries for the 61 native substances are summarized in Fig. 2. This comparison cannot distinguish if the reported signals were due to the recovery loss or due to the ion signal effect, or both. The SPE-WAX method showed decreased signals with increasing perfluorinated chain-length (i.e., C12–C18 PFCAs, and C10 and C12 PFSAs). This SPE-WAX method, using acetonitrile as elution solvent, was only suitable for C6 PFPAs and C6/C6 PFPiA among the PFPAs/PFPiAs. Both novel components of F-53B (8:2 Cl-PFESA and 6:2 Cl-PFESA) and ADONA showed good recovery performances. The results for perfluoroalkyl sulfonyl fluoride (PASF)–based compounds, except for MeFOSE and EtFOSE, ranged from 85 to 104%, and the FOSE results were about 58%. For FTCAs, FTUCAs, and FTSAs, the average signal was above 90%. However, for the total of eight PAPs, the SPE-WAX method was only suitable for 10:2 monoPAP and 6:2 diPAP. In contrast to the serum samples, for 8:2 diPAP and 6:2/8:2 diPAP, good recoveries were shown in water samples, indicating that those compounds might have a stronger affinity to proteins. The extraction efficiency for diPAPs, using the SPE-WAX method, may be improved by inhibiting the interaction between diPAPs and serum proteins.

The SPE-HLB method showed higher signals for PFSAs including long-chain PFSAs (C10, C12; see Fig. 2). A similar trend was also observed for PFPAs and PFPiAs, where the SPE-HLB method showed better signals for the three PFPAs as well as for 6:6 and 6:8 PFPiAs. The performances for novel PFECAs and PFESAs, as well as PASF-based compounds, were similar to those of the SPE-WAX method. The SPE-HLB method was suitable for the extraction of all three monoPAPs and had slightly better signals for diPAPs when compared to SPE-WAX. EtFOSA and GenX were only analyzed using the SPE-HLB method on the 4000 QTRAP, whereas moderate recoveries were observed for GenX, but EtFOSA had a recovery above 80% (see ESM Fig. S4). The observed recoveries for EtFOSA were in line with reports from previous studies [37].

Among the three extraction methods, the ion-pair extraction method was suitable for some long-chain compounds (C13, C14, C16, and C18 PFCAs, and C12 PFSA; Fig. 2). For PFPAs and PFPiAs, the ion-pair method showed better average signals (76%) for the substances investigated, except for 8:8 PFPiA. While this method performed worse with PFESAs, it showed an excellent performance for monoPAPs and diPAPs, except for 10:2 monoPAP. The ion-pair method showed the lowest performance for both PASF-based and fluorotelomer-based analytes (FTCAs, FTUCAs, and FTSAs), except for PAPs where it showed the best performance compared to both SPE methods.

The combined effect of recovery and matrix effects was similar for both SPE procedures for the majority of the PFCAs, PFSAs, PASF-based substances, PFECAs, and PFESAs. However, differences were observed for PFPAs, PFPiAs, and fluorotelomer-based substances, for which SPE-WAX performed better. In comparison, the results of the ion-pair method were noticeably lower for the PFCAs and PFSAs, likely due to matrix effects as evidenced in Table 1. This might be caused by co-extraction of matrix components and has the potential to adversely affect the sensitivity of the analysis. However, the comparatively lower recoveries of the ion-pair method are less of a problem for target analysis using isotope dilution quantification. Using authentic isotope-labeled standards compensates for any losses during extraction and matrix effects, on the premise that the instrument is sensitive enough to detect the analyte. Thus, it is less of a drawback when monitoring commonly found PFAAs, but a significant hindrance when trying to quantify compounds that lack suitable standards. In conclusion, all three extraction methods are suitable for analyzing a broad range of individual PFAS, each with specific advantages or disadvantages. Thus, the most suitable extraction method must be chosen, based on analytes of interest, under consideration of the strengths and weaknesses discussed above.

#### Placental tissue

ESM Fig. S5 shows the PFAS recoveries for the placenta I method, and ESM Fig. S6 for the placenta II method. The recoveries for 31 PFAS using the placenta I method were > 60% for all compounds, except for EtFOSA, EtFOSE, GenX, 6:2 monoPAP, and 8:2 monoPAP. The recoveries for 8:2 diPAP and 6:2/8:2 diPAP were > 200% and therefore are not shown for the placenta I method. For the placenta II method, the mean recovery for 52 PFAS was > 58%. Differences in the recoveries for both methods were observed. Firstly, probably because two different placental tissues were used and secondly, recovery differences may be due to the use of EnviCarb powder and EnviCarb cartridge in different experiments.

### Comparison of PFAAs in human serum samples using different analytical methods

PFAA concentrations in serum samples, analyzed by the ion-pair method at the MTM Research Centre and by the SPE-HLB method at the Environment Agency Austria, are shown in Table 2. Since the LOQs and LODs of the instruments of the two laboratories are different, the detection frequencies of different compounds in the samples investigated varied. Nine PFAAs were detected in the maternal serum samples, using the ion-pair method analyzed using the Waters Acquity UPLC coupled to Xevo TQ-S mass spectrometer, comprising five PFCAs (PFOA, PFNA, PFDA, PFUnDA, and PFDoDA), and four PFSAs (PFBS, PFHxS, PFHpS, and PFOS). Based on the extraction by the SPE-HLB method, followed by the analysis with the Agilent 1290 HPLC, coupled to a SCIEX 4000 QTRAP mass spectrometer, the same PFCAs and PFSAs were detected, except PFBS and PFHpS. Measured levels of PFAS in maternal serum, using either the ion-pair or the SPE-HLB method, are presented in Table 2. The concordance of the results of the eight maternal serum samples, which were analyzed using both instruments and two different extraction procedures, was 107.6% (± 21.3). No statistically significant differences between the methods used were found in the PFAS concentrations analyzed (PFOA, PFNA, PFDA, PFHxS, PFOS) (see Table 2). This confirms, in addition to the excellence of both methods, that the use of suitable mass-labeled standards is necessary for the quantification, in order to account for the recovery loss and ion signal effect [44].

**Table 2:** Maternal serum (matS) concentrations measured with two different methods using two different instruments; for the Wilcox test, the values < LOD were set at 0 and the values below the LOQ but > LOD were set at LOQ/√2

Sample	Extraction method	Instrumental method	PFOA (ng/mL)	PFNA (ng/mL)	PFDA (ng/mL)	PFHxS (ng/mL)	PFOS (ng/mL)	Wilcox test *p* value
1	Ion-pair	UPLC-MS/MS_TQ S_	0.27	0.12	0.07	0.33	0.28	0.063
	SPE-HLB	HPLC-MS/MS_QTrap_	< LOD	< LOQ	< LOQ	< LOD	< LOQ	
2	Ion-pair	UPLC-MS/MS_TQ S_	0.05	0.08	0.06	0.21	0.31	0.063
	SPE-HLB	HPLC-MS/MS_QTrap_	< LOD	< LOD	< LOQ	< LOD	< LOQ	
3	Ion-pair	UPLC-MS/MS_TQ S_	0.12	0.18	0.08	0.15	0.43	1
	SPE-HLB	HPLC-MS/MS_QTrap_	< LOD	0.21	< LOQ	< LOQ	0.48	
4	Ion-pair	UPLC-MS/MS_TQ S_	0.63	0.27	0.12	0.14	0.27	0.13
	SPE-HLB	HPLC-MS/MS_QTrap_	0.52	0.18	0.16	< LOD	< LOD	
5	Ion-pair	UPLC-MS/MS_TQ S_	0.40	0.21	0.10	0.24	0.89	1
	SPE-HLB	HPLC-MS/MS_QTrap_	0.33	0.28	0.12	< LOQ	0.96	
6	Ion-pair	UPLC-MS/MS_TQ S_	2.9	0.33	0.20	0.37	1.2	0.63
	SPE-HLB	HPLC-MS/MS_QTrap_	2.9	0.28	0.20	0.43	1.1	
7	Ion-pair	UPLC-MS/MS_TQ S_	0.52	0.18	0.07	0.13	0.30	1
	SPE-HLB	HPLC-MS/MS_QTrap_	0.46	0.27	0.08	< LOQ	< LOQ	
8	Ion-pair	UPLC-MS/MS_TQ S_	0.55	0.19	0.11	0.29	1.0	0.44
	SPE-HLB	HPLC-MS/MS_QTrap_	0.48	0.26	0.14	0.30	1.1	

### Maternal serum and cord serum samples

**Fig. 3:**
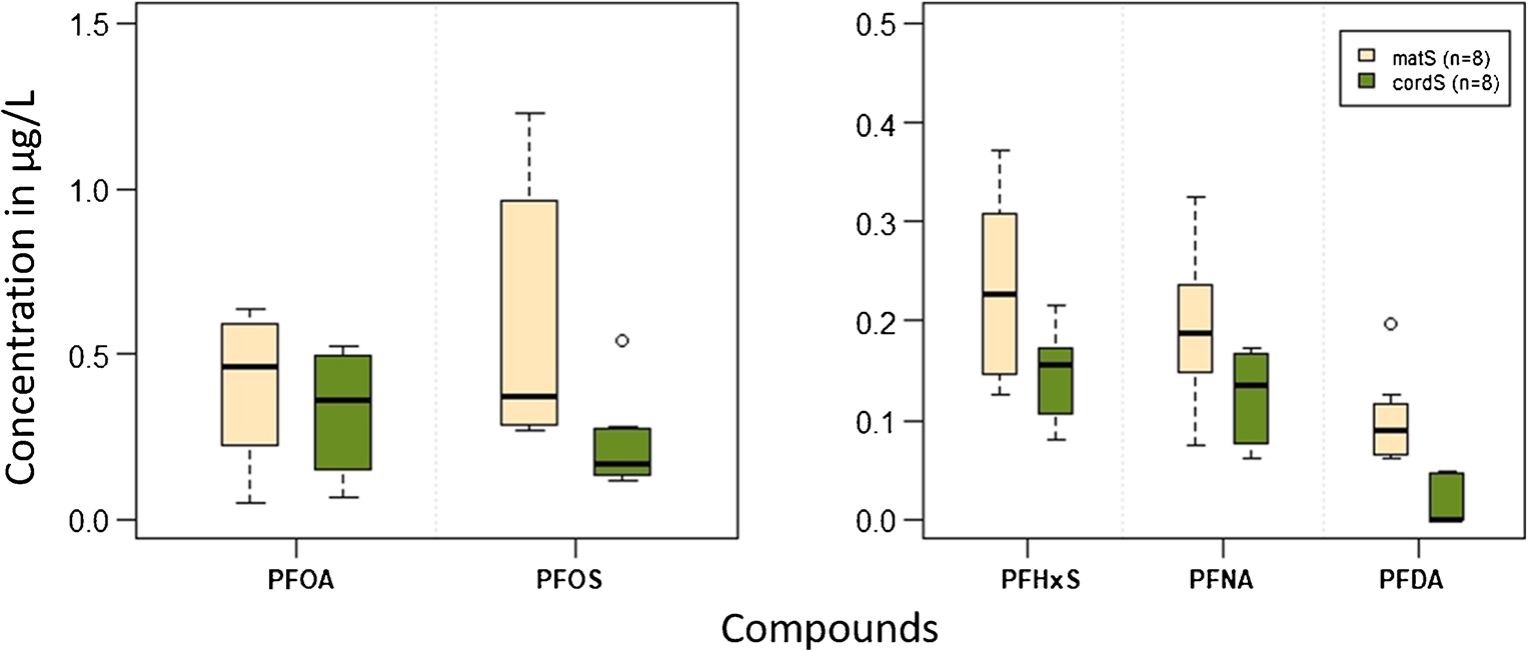
Boxplot for maternal sera and related cord sera for 5 PFAAs

There is an interest in understanding human exposure to PAPs (monoPAPs and diPAPs). Based on the results of different extraction methods, the ion-pair method was chosen to analyze corresponding cord serum samples reported in Table 2. Unfortunately, none of the PAPs showed detectable levels (< 0.12 ng/mL) in these samples; only five PFAAs were detected in the samples (see Fig. 3), with detection frequencies ranging between 69% for PFDA and 100% for PFHxS, PFOS, PFOA, and PFNA. The longer chained compounds PFUnDA and PFDoDA were not detected in the cord serum samples, but in the maternal serum samples, with detection frequencies of 75% and 38%, respectively. PFBS and PFHpS were detected in maternal and cord serum samples, with detection frequencies below 25%. Concentrations of detected PFAAs in maternal serum are shown in ESM Table S8.

The results indicate that the investigated PFAAs are able to cross the placenta barrier, which is consistent with the results of previous findings [45]. At the given low concentrations, PFUnDA and PFDoDA are likely able to cross the placental barrier to a limited extent. Both PFUnDA and PFDoDA were found in maternal serum in very low concentrations and could not be detected in umbilical cord serum. The use of larger serum volumes per individual or sample pooling could lower the limit of detection and enable the determination of more PFAS as well.

### EOF analysis in spiked bovine serum and human placental tissue

Different extraction methods resulted in different levels of EOF in the spiked samples (Table 3). The SPE-HLB method resulted in the highest average EOF concentration, followed by the ion-pair method, and then the SPE-WAX method. The EOF levels for the placenta II method were the lowest. The results with the SPE-HLB method exhibited unexpectedly high variability, which may be due to leftovers of inorganic fluorine from the sample that was not removed during the SPE washing step. This may be suspected because the SPE-WAX method had lower variability and it included an additional washing step for inorganic fluorine removal. During the ion-pair method, inorganic fluorine remains in the aqueous solution. Further optimization is needed to confirm no enrichment of inorganic fluorine when SPE-HLB is used.

**Table 3:** Extractable organic fluorine concentration (ng F/mL and ng F/g) in spiked samples, after different extraction methods

Method	SPE-HLB (ng F/mL)	SPE-WAX (ng F/mL)	Ion pair (ng F/mL)	Placenta II (ng F/g)
Matrix	Bovine serum	Bovine serum	Bovine serum	Placental tissue
Replicate I	38.6	28.8	27.1	19.8
Replicate II	53.3	29.9	35.7	25.8

Even though the levels of EOF detected by using the ion-pair method and by using the SPE-WAX method were quite similar, the composition of EOF in the respective sample extracts was different, based on the results of the recovery discussed above. The slightly higher EOF levels for the ion-pair method compared to the SPE-WAX method could be explained by the better capacity of the ion-pair method to extract longer chain PFAAs and diPAPs more efficiently. Therefore, special attention should be given when comparing the EOF levels in samples when analyzed by different extraction methods.

## Conclusion

Three different PFAS extraction procedures in serum samples were compared. The results showed that the two SPE methods are the preferred methods when considering ionization suppression and maintaining instrument sensitivity. The ion-pair method might be considered for serum samples when the analysis of PAPs is in focus as well. The presented methods for placental tissue samples worked well with the majority of PFAS investigated and were likely to be applicable to similar matrices. Nine PFAAs were detected in serum samples of eight pregnant women and seven of them in their newborns. The good data compatibility between analytical methods in the two research laboratories was achieved with the use of correct mass-labeled internal standard for quantification, as different matrix recoveries and signal ionization effects were observed for the methods evaluated in this investigation. The comparison of the extraction procedures for the EOF analysis showed that all methods revealed larger variability when compared to target PFAS analysis. As indicated above, these methods were adapted from literatures; further optimization and quality control measurements are suggested for all three methods. The EOF analysis, using combustion ion chromatography, is a promising technique that can be used for different matrices to determine the total PFAS content in a sample. It is a useful tool for regulators to address the complex issue of monitoring a large number of fluorinated chemicals at once including estimations of the presence of unidentified PFAS.

## Supplementary information

ESM 1 Details of the chemicals and analytical methods employed at the Environment Agency Austria and the MTM Research Centre as well as relevant supporting tables and figures, indicated in the main text, are available in the ESM. (PDF 836 kb).
