# Supplementary material for: Comparison of extraction methods for per- and polyfluoroalkyl substances (PFAS) in human serum and placenta samples—insights into extractable organic fluorine (EOF)
Source: Anal Bioanal Chem. 2020 Nov 19;413(3):865–76. doi: 10.1007/s00216-020-03041-5 (PMC7809006; doi:10.1007/s00216-020-03041-5)
Supplement: Supplementary file 1 — Details of the chemicals and analytical methods employed at the Environment Agency Austria and the MTM Research Centre as well as relevant supporting tables and figures, indicated in the main text, are available in the ESM. (PDF 836 kb). [file 216_2020_3041_MOESM1_ESM.pdf]

Analytical and Bioanalytical Chemistry

Electronic Supplementary Material

Comparison of extraction methods for per- and polyfluoroalkyl substances (PFAS) in human serum and placenta samples – insights into extractable organic fluorine (EOF)

Andreas-Marius Kaiser, Rudolf Aro, Anna Kärrman, Stefan Weiss, Christina Hartmann, Maria Uhl, Martin Forsthuber, Claudia Gundacker, Leo W.Y Yeung

Table of Contents

P.2 Details of the chemicals and analytical methods employed in Environment Agency Austria
P.5 Details of the chemicals and analytical methods employed in MTM Research Centre
P.35 Statistically significant correlations (Spearman´s correlation coefficients, r) between the concentrations of PFOA and other long-chain PFCAs in the maternal serum samples
P.37 Abbreviations
P.41 References

P.4 Fig. S1. The LC gradient used at the Environment Agency Austria for PFAS analysis
P.8 Fig. S2: The LC gradient used at the MTM for PFAS analysis
P.9 Fig. S3: Elements of the total fluorine content in one sample
P.10 Fig. S4: Recoveries [%] for 31 PFAS using the SPE-HLB method and the 4000 QTRAP – three quality control standards (bovine serum spiked with 10 µL of native standard mix I (see Table S1)) from three different batches (three different days) were used.
P.11 Fig. S5: Recoveries [%] in the placental tissue samples for placenta I method.
P.11 Fig. S6: Recoveries [%] in the placental tissue samples for placenta II method

P.12 Table S1: Standards used for the calibration, quality controls and samples (internal standards)
P.12 Table S2: Summary of the applied extraction procedures and used instruments.
P.13 Table S3: The analyzed PFAS related to their specific sub classes
P.18 Table S4: Internal Standards (IS) and Recovery Standards (RS) for a) 4000 QTRAP and b) TQ-S (and TQ-S µ for ADONA and GenX).
P.21 Table S5: Blanks used for quality controls are reported for 500 µL MilliQ water for each extraction method and for both instruments a) 4000 QTRAP and b) TQ-S and TQ-S micro (ADONA and GenX).
P.25 Table S6: The analytical parameters for the 4000 QTRAP
P.28 Table S7: The analytical parameters for the TQ-S and TQ-S micro
P.34 Table S8: Descriptive statistics for maternal serum samples (n=8).
P.36 Table S9: Evaluation of inorganic fluorine elimination of different extraction methods with different matrices

Environment Agency Austria

Chemicals. For sample preparation, methanol (Pestinorm®, VWR International, Leuven, Belgium) and acetonitrile (Optigrade, Promochem, LGC Standards, Wesel, Germany) were distilled before use. Filtered tap water (fTW) was prepared by filtering with Oasis HLB-column (6 mL, 500 mg) cartridges (Waters Corporation, Milford, MA, USA). LC-MS-grade methanol and LC-MS-grade water was purchased from Merck (Darmstadt, Germany) and ammonium acetate for MS from Sigma-Aldrich® (St. Louis, MO, USA). Formic acid (98-100%) was purchased from Merck (Darmstadt, Germany) and ammonia solution (25%) from Fisher Scientific (Loughborough, UK). Supeclean™ LC-18 SPE Bulk, adult bovine serum was supplied by Sigma-Aldrich® (St. Louis, MO, USA), Supelclean ENVI-Carb 120/400 by Supelco (Bellefonte, USA) and n-hexane by Pestinorm® (VWR International, Leuven, Belgium).

Serum samples. The solid phase extraction using hydrophilic-lipophilic balance sorbent (SPE-HLB) based on a published method by Kuklenyik et al. (2004) [1] with modifications was applied. In brief, 500 µL of serum sample was transferred into a polypropylene (PP) tube and spiked with 10 ng (each compound) of an internal standard mix I, 0.5 µg/L each compound (see Table S1). Samples were vortexed before and after adding 3 mL of a 0.1 M formic acid (HFA) solution, then ultrasonicated for 20 min followed by SPE-HLB extraction. The schematics and details of the SPE-HLB procedures are provided in Fig. 1. The HLB-cartridges were conditioned with 6 mL methanol (MeOH) and equilibrated with 6 mL of the 0.1 M HFA solution in fTW. After the samples were loaded onto the cartridge, they were washed with 6 mL of 0.1 M HFA solution in fTW, followed by 6 mL of HFA solution containing 40% of MeOH, and finally 1.5 mL of 0.01% NH_4OH solution in fTW.

The cartridges were dried under a nitrogen flow (≤0.4 bar) until dryness. The analytes were then eluted into a PP-tube using 6 mL acetonitrile (ACN) containing 0.0001% NH_4OH. All steps were conducted without the use of a vacuum pump to allow enough contact time during extraction. The ACN extract volume was reduced to 500 µL under a mild nitrogen flow at 40 °C and then adjusted to 1 mL with a 20 mM acetic acid (HAC) solution in fTW. The samples were transferred into PP-vials for instrumental analysis.

Instrumental analysis. The target PFAS analysis in serum and placental tissue samples was performed by high-performance liquid chromatography tandem-mass spectrometry (HPLC-MS/MS). This analytical system was composed of an Agilent Technologies 1290 Infinity Series (Agilent Technologies, Santa Clara, CA, USA) HPLC and a SCIEX 4000 QTRAP mass spectrometer (AB Sciex Technologies, Framingham, MA, USA) in negative electrospray ionization (ESI) mode. The analytical column was a Luna 5 µm C18(2), 100 x 2 mm (Phenomenex, California, USA). Eluents were methanol (mobile phase B) and LC-MS-grade water containing 10 mM ammonium acetate (mobile phase A). Details of the LC gradient are given in Fig. S1. The gradient (mobile phase B) was held linear at 5% for 3 min, increased to 35% at 8 min, 60% at 18 min, 95% at 20 min and then changed to the initial conditions. The flow rate was 0.3 mL/min, the analyzing time 23 min, the injection volume 10 µL and the column temperature was 40 °C. The first 4.5 min and the last 4 min went directly to the waste to preserve the column and to diminish the effect on the sensitivity of the instrument.

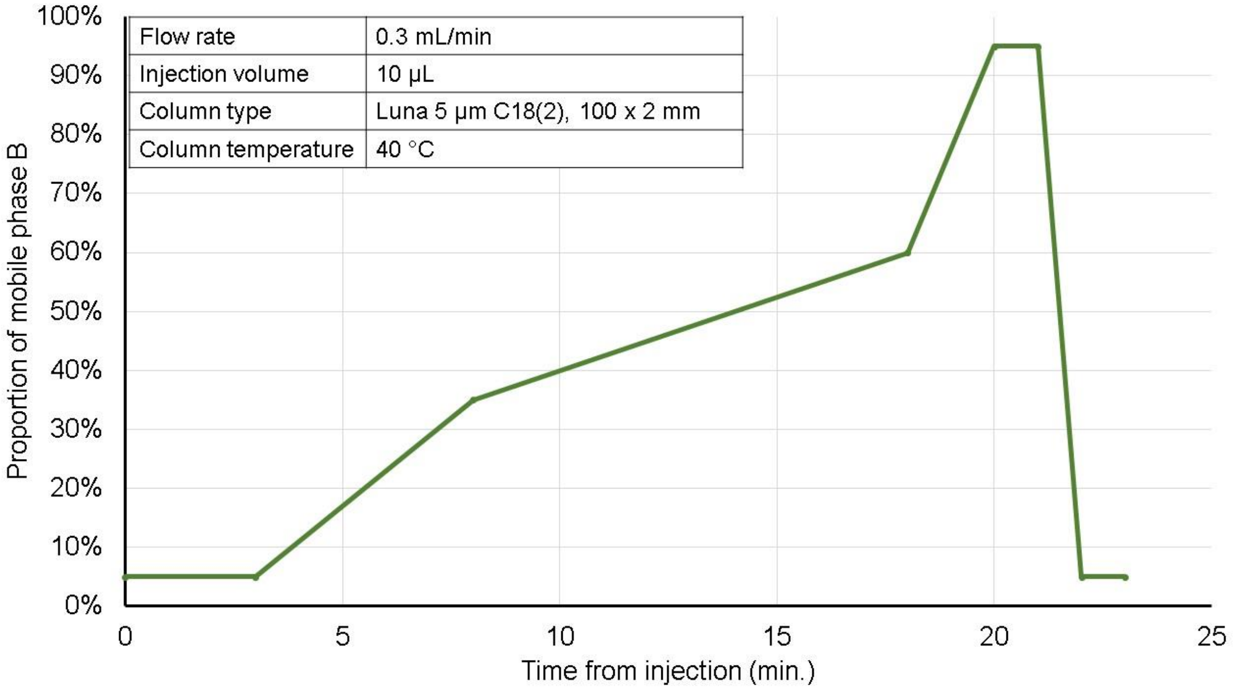

Fig. S1 The LC gradient used at the Environment Agency Austria for PFAS analysis

Quality assurance and control (QA/QC) measures. One quality control (QC) and one blank sample were prepared and measured on the same day. Each QC sample consisted of 500 µL bovine serum spiked with 10 ng (each compound) of the internal standard mix I (0.5 µg/mL) and 5 ng (each compound) of a native standard mix I (0.5 µg/mL) (see Table S1). For the blank 500 µL fTW were used containing 10 ng (each compound) of the internal standard mix I (0.5 µg/mL). One QC and one blank were extracted within each sample batch. Limit of detections (LODs) and limit of quantifications (LOQs) were calculated in accordance with DIN 32645 (see Table S3). Calibration curves included ten concentration points ranging between 0.05 and 25 ng/mL. In general, the transition with the highest intensity was used, except for PFOS where interferences with taurodeoxycholic acid could lead to an overestimation [2].

MTM Research Centre

Chemicals. Ammonium acetate (NH_4OAc), tert-butyl methyl ether (MTBE) (purity ≥99.8%), tetrabutylammonium bisulfate (TBA) with a purity of ≥ 99% and n-methylpiperidine (1-MP, 99%) were purchased from Sigma-Aldrich® (St. Louis, MO, USA). Supeclean™ ENVI-Carb™ SPE Tubes (6 mL, 250 mg) were supplied by Supelco (Bellefonte, PA, USA), and OASIS® WAX (6 mL, 150 mg) and OASIS® HLB (6 mL, 200 mg) by Waters Corporation (Milford, MA, USA). Formic Acid (98/100%), methanol HPLC-grade (≥ 99.8%), methanol LC-MS-grade (≥ 99.9%), acetonitrile HPLC-grade (≥ 99.9%) and ammonium solution (25%) were purchased from Fisher Scientific (Pittsburgh, PA, USA). As quality control material to check for inorganic fluorine contamination a multielement ion chromatography anion standard solution by Sigma-Aldrich® (St. Louis, MO, USA) was used.

Target PFAS include PFBA, PFPeA, PFHxA, PFHpA, PFOA, PFNA, PFDA, PFUnDA, PFDoDA, PFTrDA, PFTeDA, PFHxDA, PFOcDA, PFBS, PFPeS, PFHxS, PFHpS, PFOS, PFNS, PFDS, PFDoDS, perfluorobutyl sulfonamide (FBSA), N-methyl-nonafluorobutane sulfonamide (MeFBSA), perfluoro-1-hexane sulfonamide (FHxSA), N-methyl-perfluoro-1-hexane sulfonamide (MeFHxSA), FOSA, MeFOSA, EtFOSA, MeFOSE, EtFOSAA, MeFOSAA, EtFOSE, 4:2 FTSA, 6:2 FTSA, 8:2 FTSA, 10:2 FTSA, 3-perfluoropropyl propanoic acid (3:3 FTCA), 3-perfluoropentyl propanoic acid (5:3 FTCA), 3-perfluoroheptyl propanoic acid (7:3 FTCA), 2H-perfluoro-2-octenoic acid (6:2 FTUCA), 2H-perfluoro-2-decenoic acid (8:2 FTUCA), 2H-perfluoro-2-dodecenoic acid (10:2 FTUCA), 6:2 polyfluoroalkyl phosphoric acid monoester (6:2 monoPAP), 8:2 polyfluoroalkyl phosphoric acid monoester (8:2 monoPAP), 10:2 polyfluoroalkyl

phosphoric acid monoester (10:2 monoPAP), 6:2 diPAP, 8:2 diPAP and 6:2/8:2 diPAP and 10:2 diPAP, (bis-)2-N-ethylperfluorooctane-1-sulfonamido-ethyl-phospate (diSAmPAP), ADONA, HFPO-DA (GenX), 6:2 chlorinated polyfluorinated ether sulfonate (6:2 Cl-PFESA), 8:2 chlorinated polyfluorinated ether sulfonate (8:2 Cl-PFESA), perfluorohexyl phosphonic acid (PFHxPA), perfluorooctyl phosphonic acid (PFOPA), perfluorodecyl phosphonic acid (PFDPA), 6:6 bis-perfluorohexyl phosphinate (6:6 PFPiA), perfluorohexyl-perfluorooctyl phosphinate (6:8 PFPiA), bis-perfluorooctyl phosphinate (8:8 PFPiA), and perfluoro-4-ethylcyclohexane sulfonate (PFECHS). Analytical standards were purchased from Wellington Laboratories (Ontario, Canada) with a purity ≥96, and Apollo Scientific Ltd (FBSA) with a purity ≥97%.

SPE-WAX. Solid phase extraction with weak anion exchange sorbent (SPE-WAX) methods published by Kuklenyik et al. (2004) [1] and Miyake et al. (2007) [3] with modifications was used. Before the extraction procedure, the 500 µL serum sample was spiked with 2 ng (each compound) of the internal standard mix II (see Table S1). The samples (500 µL) were transferred to PP-tubes and were vortexed before and after the addition of 6 mL 0.1 M HFA solution in MilliQ water and subsequently ultrasonicated for 15 min. The WAX-cartridges were conditioned with 6 mL MeOH containing 0.1% NH_4OH, then washed with 6 mL MeOH and were equilibrated with 6 mL of the 0.1 M HFA solution in MilliQ water. After the samples were loaded on the WAX-cartridges, they were washed with 6 mL of 0.1 M HFA solution, 6 mL of 0.1 M HFA solution containing 40% of MeOH and 18 mL MilliQ water containing 0.01% NH_4OH to remove inorganic fluorine. To remove any residual water, the cartridges were centrifuged at 4630 g for 15 seconds, then the analytes were eluted with 0.1% NH_4OH in 6 mL ACN. The collected ACN extract was

reduced to 200 µL in the vacuum-evaporator (60°C and 250 mbar). Finally, the 200 µL were split into two vials, one with 40% and the other with 80% organic solvent content for different PFAS analysis (see Fig. 1).

Ion-pair method. A modified ion-pair method published by Hansen et al. (2001) [4] was used. 500 µL serum sample was spiked with 2 ng (each compound) of the internal standard mix II (see Table S1). To each sample, 2 mL 0.5 M TBA in MilliQ-water and 5 mL of MTBE were added. The sample was shaken for 15 min, centrifuged for 10 min at 8,000 g and then the supernatant was transferred into a new PP-tube. After that, 3 mL of MTBE were added and the extraction step was repeated twice. Supernatants from the three cycles were combined in the same PP-tube, evaporated to 0.2 mL, adjusted to 1 mL with MeOH and evaporated again to 0.2 mL to remove the MTBE content. Finally, the 200 µL were split into two vials, one with 40% and the other with 80% organic solvent content for different PFAS analysis (see Fig. 1).

Instrumental analysis. Target PFAS analysis were performed with an ultra-performance liquid chromatography (UPLC) system from Waters (Acquity UPLC®, Waters Corporation, Milford, MA, USA) coupled to either a Xevo TQ-S or a Xevo TQ-S-micro mass spectrometer in ESI negative mode. The analytical column was an ACQUITY UPLC® BEH C18 1.7 µm, 2.1 x 100 mm column (Waters Corporation, Milford, MA, USA), and eluents for the mobile phases were a 70:30-mixture of MilliQ-water and methanol (mobile phase A) and methanol (mobile phase B), both containing 2 mmol/L ammonium acetate and 5 mmol/L n-methylpiperidine (only TQ-S). The column temperature was 50 °C, the flow rate and the injection volume were 0.3 mL/min and 10 µL, respectively. Details of the LC

gradient are given in Fig. S2. The analysis time was 17 min; the initial condition was mobile phase B at 1%, kept constant until 0.57 min and then increased linearly to 100% by 13 min. Then mobile phase B was held at 100% for 1 minute and thereafter decreased to 1% by 14.2 minutes after which the column was allowed to equilibrate until minute 17.

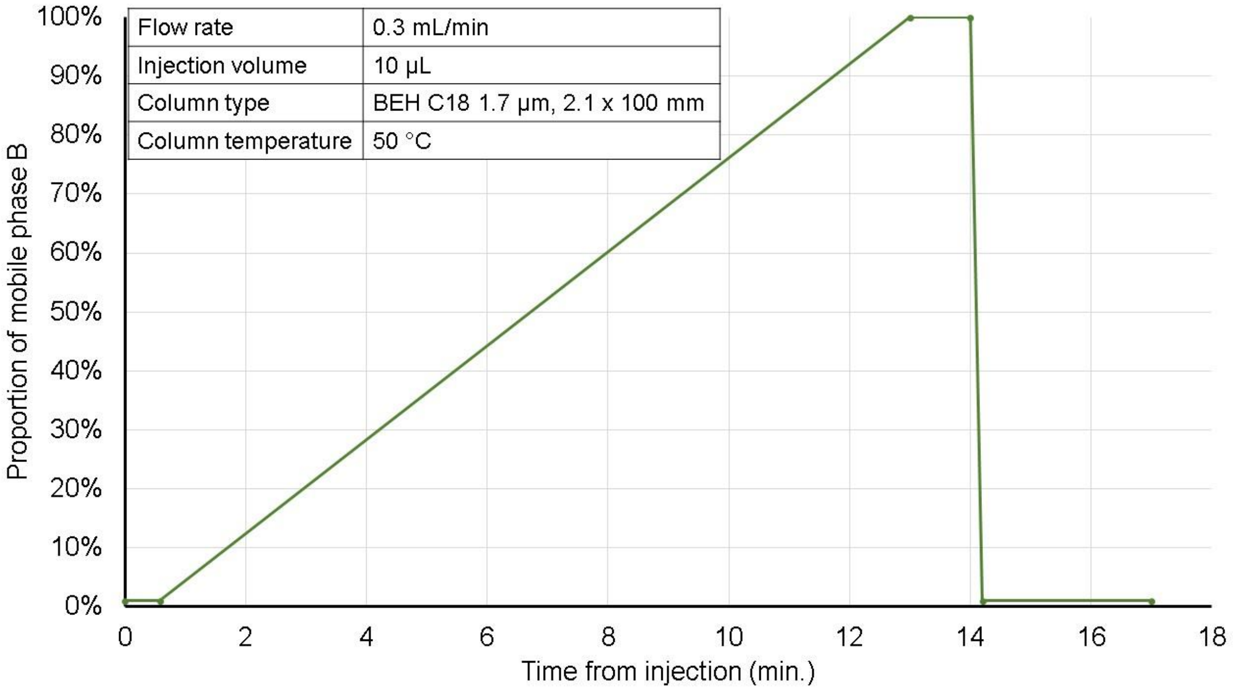

Fig. S2 The LC gradient used at the MTM for PFAS analysis

Extractable organic fluorine (EOF) analysis. Combustion ion chromatography (CIC) was used to analyze the EOF content (see Fig. S3) in the samples. The instrument consisted of a combustion module (Analytik Jena, Germany), a 920 Absorber Module and a 930 Compact IC Flex ion chromatograph (Metrohm, Switzerland). The ion exchange column was a Metrosep A Supp 5 – 150/4.0 (Metrohm, Switzerland), the eluent was an a carbonate buffer (64 mmo/L sodium carbonate and 20 mmol/L sodium bicarbonate) from Sigma-Aldrich®, isocratic elution was used. 100 µL of the sample was placed into the

furnace using a quartz boat. The liquid sample was oxidized at high temperature (900–1050 °C) and converted into hydrogen fluoride (HF), which was absorbed in MilliQ water. After an aliquot of the absorber solution was injected, the fluoride content was determined by conductivity.

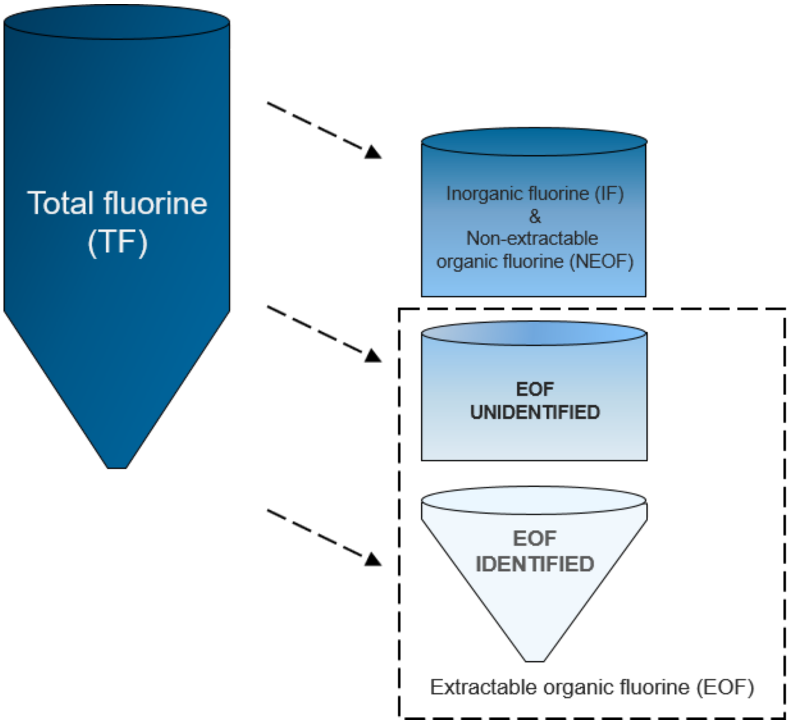

Fig. S3 Elements of the total fluorine content in one sample

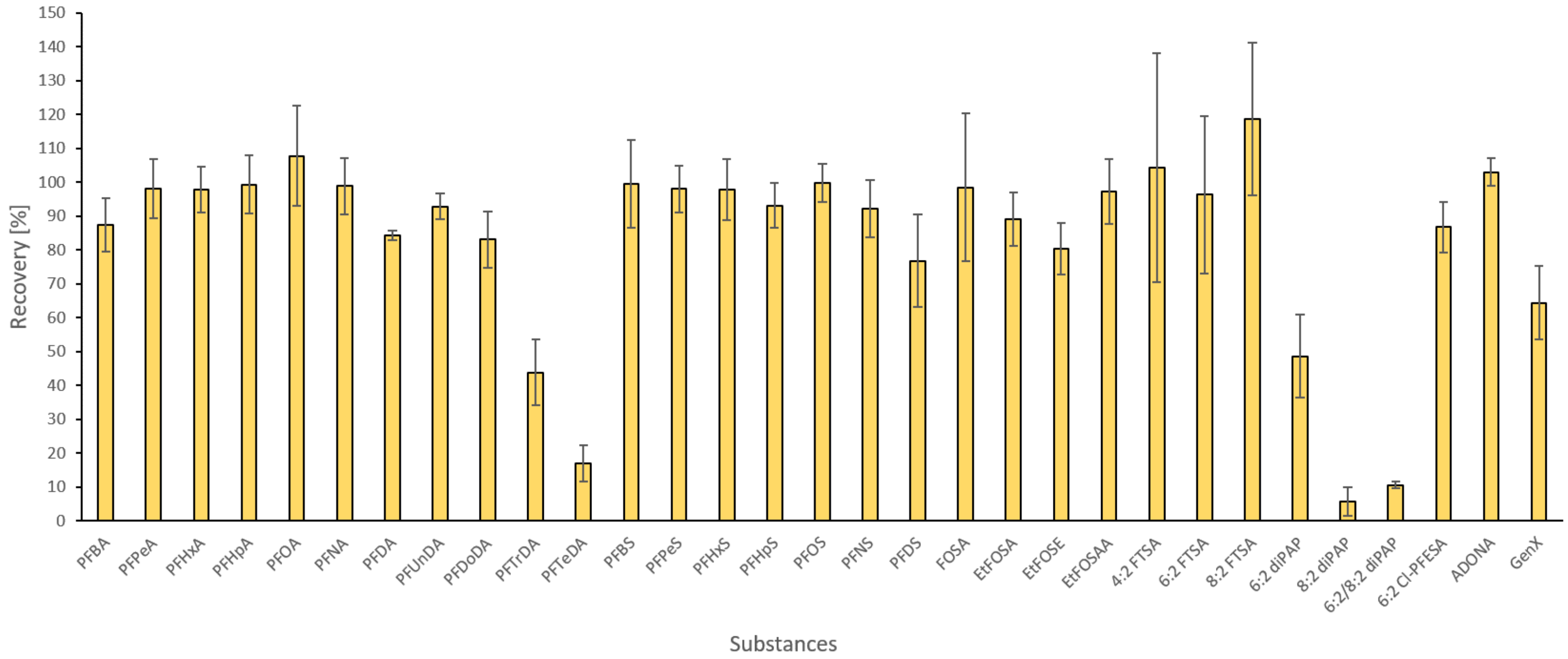

Fig. S4 Recoveries [%] for 31 PFAS using the SPE-HLB method and the 4000 QTRAP – three quality control standards (bovine serum spiked with 10 µL of native standard mix I (see Table S1)) from three different batches (three different days) were used

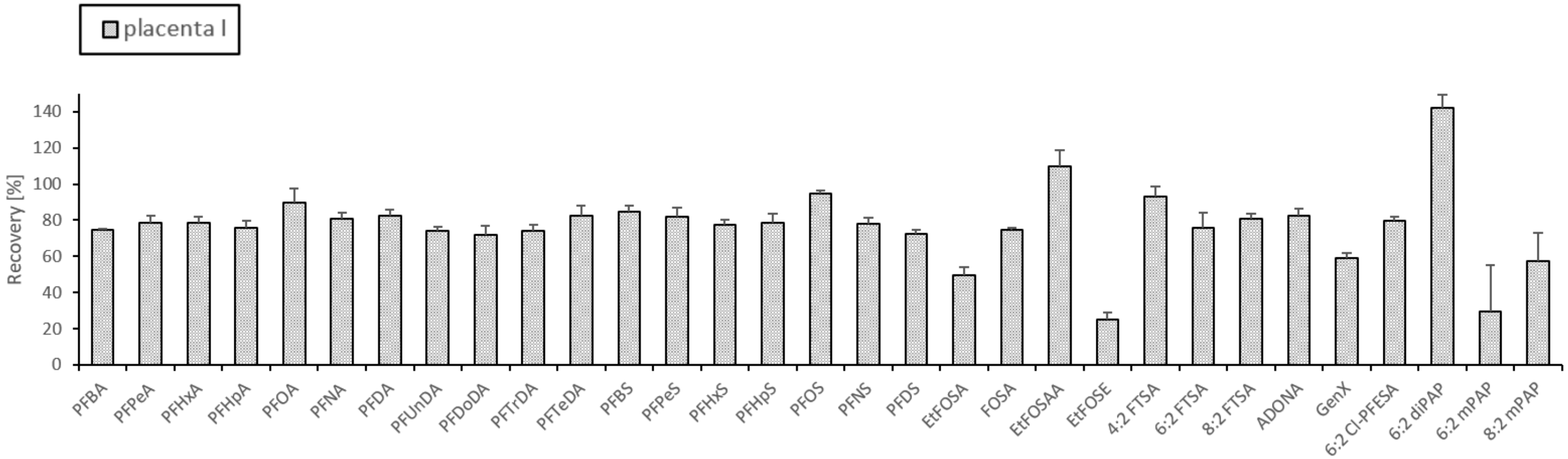

Fig. S5 Recoveries [%] in the placental tissue samples for placenta I method

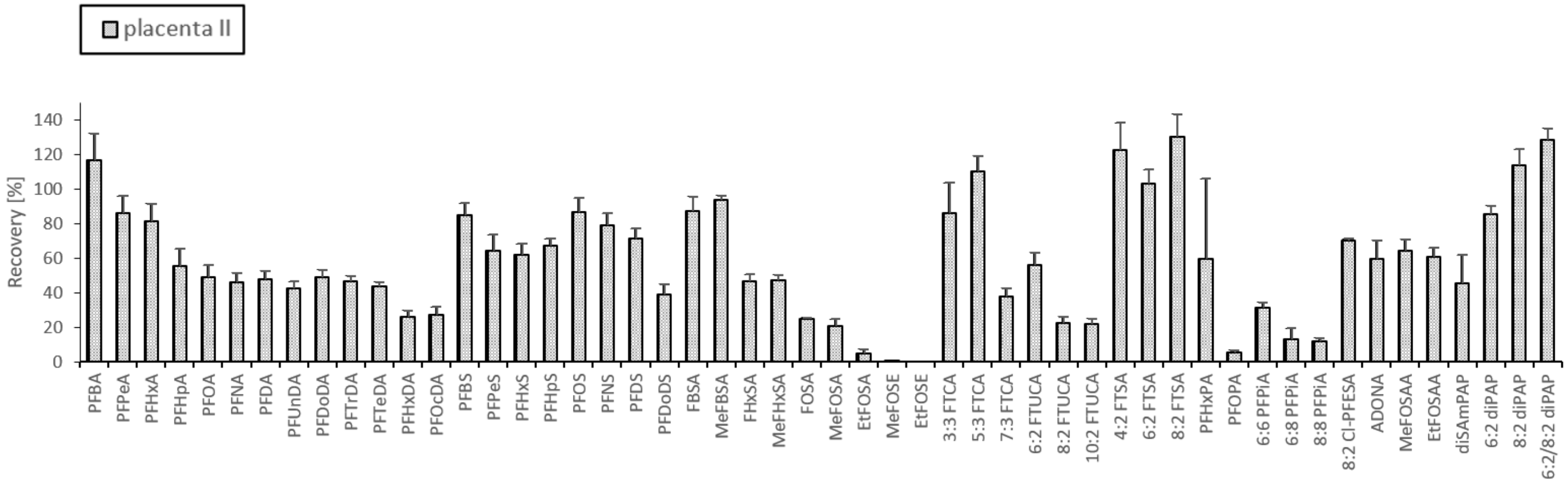

Fig. S6 Recoveries [%] in the placental tissue samples for placenta II method

Table S1 Standards used for the calibration, quality controls and samples (internal standards)

**Standard**	**Method and compounds**
**native standard mix I**	**SPE-HLB, placenta I method**<br>PFCA (C4-C14), PFSA (C4-C10), PFOSA, N-Et-PFOSA, N-Et-FOSAA, N-Et-FOSE, 4:2 FTSA, 6:2 FTSA, 8:2 FTSA, 6:2 Cl-PFESA (F-53B), HFPO-DA (GenX), ADONA, 6:2 diPAP, 8:2 diPAP and 6:2/8:2 diPAP; (6:2 mPAP and 8:2 mPAP)
**internal standard mix I**	**SPE-HLB, placenta I method**<br>isotopes from PFCA (C4, C6, C8-C12), PFSA (C6 and C8), EtFOSA, EtFOSAA, EtFOSE, 6:2 FTSA and 8:2 diPAP
**native standard mix II**	**SPE-WAX, SPE-HLB, ion-pair, placenta II method**<br>all substances shown in Table S3
**internal standard mix II**	**SPE-WAX, SPE-HLB, ion-pair, placenta II method**<br>all substances shown in Table S3

Table S2 Summary of the applied extraction procedures and used instruments

**Extraction procedure**	**Instrument**	**Sample quantity**
SPE-HLB, placenta I method	(a) HPLC-MS/MS (AB Sciex 4000 QTRAP)	8 x maternal serum<br>1 x placental tissue
SPE-WAX, SPE-HLB, ion-pair, placenta II method<br>SPE-WAX, SPE-HLB, ion-pair, placenta II method	(b) UPLC-MS/MS (Waters Xevo TQ-S)<br>(c) UPLC-MS/MS (Waters Xevo TQ-S micro)	8 x maternal serum<br>8 x cord serum<br>1 x placental tissue
SPE-WAX, SPE-HLB, ion-pair, placenta II method	(d) Combustion ion chromatography	bovine serum<br>MilliQ-water<br>placental tissue

Table S3 The analyzed PFAS related to their specific sub classes (perfluoroalkyl carboxylic acids (PFCAs), perfluoroalkane sulfonic acids (PFSAs), perfluoro phosphonic acids (PFPA), perfluoroalkyl phosphinic acids (PFPiA), perfluoroether carboxylic and sulfonic acids (PFECA and PFESA), PASF-based substances and fluorotelomer-based substances) - PFECHS* is part of the PASF-based substances in this table for reasons of simplicity, to be precise it would account to another sub class, the cyclic perfluoroalkyl acids – limits of detection (LODs) and limits of quantification (LOQs) are shown for both instruments a) 4000 QTRAP and b) TQ-S (and TQ-S micro for ADONA and GenX). When at least two product ions were monitored for an analyte, the results were only reported if both were detected and their ratio was within 50 % of the ratio observed in a standard sample

Sub-class	Abbreviation	Compound name	CAS number	Molar mass (g/mol)	Molecular formula	LODs [ng/mL]<br>a) 4000 QTRAP<br>b) TQ-S	LOQs [ng/mL]<br>a) 4000 QTRAP<br>b) TQ-S
PFCA	PFBA	perfluoro-n-butanoic acid	375-22-4	214.04	$C_4HF_7O_2$	a) 0.25<br>b) 0.090	a) 0.50<br>b) 0.18
	PFPeA	perfluoro-n-pentanoic acid	2706-90-3	264.05	$C_5HF_9O_2$	a) 0.42<br>b) 0.050	a) 0.84<br>b) 0.10
	PFHxA	perfluoro-n-hexanoic acid	307-24-4	314.05	$C_6HF_{11}O_2$	a) 0.060<br>b) 0.050	a) 0.12<br>b) 0.10
	PFHpA	perfluoro-n-heptanoic acid	375-85-9	364.06	$C_7HF_{13}O_2$	a) 0.075<br>b) 0.070	a) 0.15<br>b) 0.14
	PFOA	perfluoro-n-octanoic acid	335-67-1	414.07	$C_8HF_{15}O_2$	a) 0.16<br>b) 0.060	a) 0.32<br>b) 0.12
	PFNA	perfluoro-n-nonanoic acid	375-95-1	464.08	$C_9HF_{17}O_2$	a) 0.090<br>b) 0.015	a) 0.18<br>b) 0.030
	PFDA	perfluoro-n-decanoic acid	335-76-2	514.08	$C_{10}HF_{19}O_2$	a) 0.040<br>b) 0.020	a) 0.080<br>b) 0.040
	PFUnDA	perfluoro-n-undecanoic acid	2058-94-8	564.09	$C_{11}HF_{21}O_2$	a) 0.070<br>b) 0.0275	a) 0.14<br>b) 0.055
	PFDoDA	perfluoro-n-dodecanoic acid	307-55-1	614.10	$C_{12}HF_{23}O_2$	a) 0.040<br>b) 0.025	a) 0.080<br>b) 0.050

	PFTrDA	perfluoro-n-tridecanoic acid	72629-94-8	664.11	$C_{13}HF_{25}O_2$	a) 0.225<br>b) 0.030	a) 0.45<br>b) 0.060
	PFTeDA	perfluoro-n-tetradecanoic acid	376-06-7	714.11	$C_{14}HF_{27}O_2$	a) 0.80<br>b) 0.030	a) 1.6<br>b) 0.060
	PFHxDA	perfluoro-n-hexadecanoic acid	67905-19-5	814.13	$C_{16}HF_{31}O_2$	b) 0.20	b) 0.40
	PFOcDA	perfluoro-n-octadecanoic acid	16517-11-6	914.11	$C_{18}HF_{35}O_2$	b) 10	b) 20

**PFSA**	PFBS	perfluoro-n-butane sulfonate	375-73-5	300.10	$C_4HF_9SO_3$	a) 0.15<br>b) 0.0060	a) 0.30<br>b) 0.012
	PFPeS	perfluoro-n-pentane sulfonate	630402-22-1	349.10	$C_5F_{11}SO_3$ (Na)	a) 0.75<br>b) 0.0060	a) 1.5<br>b) 0.012
	PFHxS	perfluoro-n-hexane sulfonate	355-46-4	400.12	$C_6HF_{13}SO_3$	a) 0.12<br>b) 0.040	a) 0.24<br>b) 0.080
	PFHpS	perfluoro-n-heptane sulfonate	375-92-8	450.12	$C_7HF_{15}SO_3$	a) 0.25<br>b) 0.010	a) 0.50<br>b) 0.020
	PFOS	perfluoro-n-octane sulfonate	1763-23-1	500.13	$C_8HF_{17}SO_3$	a) 0.225<br>b) 0.050	a) 0.45<br>b) 0.10
	PFNS	perfluoro-n-nonane sulfonate	98789-57-2	549.13	$C_9F_{19}SO_3$ (Na)	a) 0.55<br>b) 0.050	a) 1.1<br>b) 0.10
	PFDS	perfluoro-n-decane sulfonate	335-77-3	600.15	$C_{10}HF_{21}SO_3$	a) 0.20<br>b) 0.050	a) 0.40<br>b) 0.10
	PFDoDS	perfluoro-n-dodecansulfonate	343629-43-6	699.15	$C_{12}F_{25}SO_3$	b) 1.0	b) 2.0

**PFPA**	PFHxPA	perfluorohexyl phosphonic acid	40143-76-8	400.03	$C_6H_2F_{13}PO_3$	b) 0.050	b) 0.10
	PFOPA	perfluorooctyl phosphonic acid	40143-78-0	500.05	$C_8H_2F_{17}PO_3$	b) 0.20	b) 0.40

	PFDPA	perfluorodecyl phosphonic acid	52299-26-0	600.06	$C_{10}H_2F_{21}PO_3$	b) 0.010	b) 0.020
PFPiA	6:6 PFPiA	bis-perfluorohexyl phosphinate	70609-44-8	701.06	$C_{12}F_{26}O_2P$ (Na)	b) 0.050	b) 0.10
	6:8 PFPiA	Perfluorohexyl-perfluorooctyl phosphinate	not available	801.07	$C_{14}F_{30}O_2P$	b) 2.5	b) 5.0
	8:8 PFPiA	bis-perfluorooctyl phosphinate	500776-69-2	901.09	$C_{16}F_{34}O_2P$ (Na)	b) 10	b) 20
PFECA and PFESA	ADONA	dodecalfluoro-3H-4,8-dioxanonanoate	958445-44-8	376.96	$C_7HF_{12}O_4$ (Na)	a) 0.50<br>b) 0.025	a) 1.0<br>b) 0.050
	HFPO-DA (GenX)	hexafluoropropylene oxide-dimer acid	13252-13-6	330.05	$C_6HF_{11}O_3$	a) 1.4<br>b) 0.025	a) 2.8<br>b) 0.050
	6:2 Cl-PFESA (major F-53B)	6:2 chlorinated polyfluorinated ether sulfonate	73606-19-6	531.57	$C_8F_{16}ClSO_4$ (K)	a) 1.0<br>b) 0.010	a) 2.0<br>b) 0.020
	8:2 Cl-PFESA (minor F-53B)	8:2 chlorinated polyfluorinated ether sulfonate	83329-89-9	631.59	$C_{10}F_{20}ClSO_4$ (K)	b) 0.010	b) 0.020
PASF-based substances	PFECHS*	perfluoro-4-ethylcyclohexane sulfonate	67584-42-3	461.12	$C_8F_{15}SO_3$ (K)	b) 0.010	b) 0.020
	FBSA	perfluoro-n-butane sulfonamide	30334-69-1	299.12	$C_4H_2F_9NO_2S$	b) 0.25	b) 0.50
	MeFBSA	N-methyl-nonafluoro-n-butane sulfonamide	68298-12-4	313.14	$C_5H_4F_9NO_2S$	b) 0.25	b) 0.50
	PFOSA	perfluoro-n-octane sulfonamide	754-91-6	499.15	$C_8H_2F_{17}NO_2S$	a) 0.235<br>b) 0.020	a) 0.47<br>b) 0.040
	MeFOSA	N-methyl-perfluoro-n-octane sulfonamide	31506-32-8	513.17	$C_9H_4F_{17}NO_2S$	b) 0.75	b) 1.2

	EtFOSA	N-ethyl-perfluoro-n-octane sulfonamide	4151-50-2	527.20	$C_{10}H_6F_{17}NO_2S$	a) 0.24<br>b) 0.60	a) 0.48<br>b) 1.2
	MeFOSE	N-methyl-perfluoro-n-octane sulfonamido-ethanol	24448-09-7	557.23	$C_{11}H_8F_{17}NO_3S$	b) 0.60	b) 1.2
	EtFOSE	N-ethyl-perfluoro-n-octane sulfonamidoethanol	1691-99-2	571.25	$C_{12}H_{10}F_{17}NO_3S$	a) 0.475<br>b) 0.60	a) 0.95<br>b) 1.2
	MeFOSAA	N-methyl-perfluoro-n-octane sulfonamido acetic acid	2355-31-9	571.21	$C_{11}H_6F_{17}NO_4S$	b) 0.025	b) 0.050
	EtFOSAA	N-ethyl-perfluoro-n-octane sulfonamido acetic acid	2991-50-6	585.24	$C_{12}H_8F_{17}NO_4S$	b) 0.10	b) 0.20
	FHxSA	perfluoro-n-hexane sulfonamide	41997-13-1	399.13	$C_6H_2F_{13}NO_2S$	b) 0.025	b) 0.050
	MeFHxSA	N-methyl-perfluoro-n-hexane sulfonamide	68259-15-4	413.16	$C_7H_4F_{13}NO_2S$	b) 0.15	b) 0.30

**Fluorotelomer-based substances**	3:3 FTCA	3-perfluoropropyl propanoic acid	356-02-5	242.09	$C_6H_5F_7O_2$	b) 0.050	b) 0.10
	5:3 FTCA	3-perfluoropentyl propanoic acid	914637-49-3	342.11	$C_8H_5F_{11}O_2$	b) 0.025	b) 0.050
	7:3 FTCA	3-perfluoroheptyl propanoic acid	812-70-4	442.12	$C_{10}H_5F_{12}O_2$	b) 0.025	b) 0.050
	6:2 FTUCA	2H-perfluoro-2-octenoic acid	261503-40-6	358.08	$C_8H_2F_{12}O_2$	b) 0.025	b) 0.050
	8:2 FTUCA	2H-perfluoro-2-decenoic acid	147874-76-8	458.10	$C_{10}H_2F_{16}O_2$	b) 0.025	b) 0.050
	10:2 FTUCA	2H-perfluoro-2-dodecenoic acid	not available	558.11	$C_{12}H_2F_{20}O_2$	b) 0.050	b) 0.10
	4:2 FTSA	4:2 fluorotelomer sulfonate (1H,1H,2H,2H-perfluorohexane sulfonate)	757124-72-4	328.15	$C_6H_5F_9SO_3$	a) 0.30<br>b) 0.010	a) 0.60<br>b) 0.020
	6:2 FTSA	6:2 fluorotelomer sulfonate (1H,1H,2H,2H-perfluorooctane sulfonate)	27619-97-2	428.17	$C_8H_5F_{13}SO_3$	a) 0.665<br>b) 0.010	a) 1.3<br>b) 0.020
	8:2 FTSA	8:2 fluorotelomer sulfonate (1H,1H,2H,2H-perfluorodecane sulfonate)	39108-34-4	528.18	$C_{10}H_5F_{17}SO_3$	a) 0.65<br>b) 0.010	a) 1.3<br>b) 0.020
	10:2 FTSA	10:2 fluorotelomer sulfonate (1H,1H,2H,2H-perfluorododecane sulfonate)	120226-60-0	628.20	$C_{12}H_5F_{21}SO_3$	b) 0.25	b) 0.50

	diSAmPAP	(bis-)2-N-ethylperfluorooctane-1-sulfonamido-ethyl-phospate	not available	1203.46	$C_{24}H_{18}F_{34}N_2O_8PS_2$	b) 0.15	b) 0.30
	6:2 monoPAP	6:2 polyfluoroalkyl phosphoric acid monoester	57678-01-0	444.08	$C_8H_6F_{13}O_4P$	b) 1.0	b) 2.0
	8:2 monoPAP	8:2 polyfluoroalkyl phosphoric acid monoester	57678-03-3	542.08	$C_{10}H_4F_{17}O_4P$	b) 0.25	b) 0.50
	10:2 monoPAP	10:2 polyfluoroalkyl phosphoric acid monoester	57678-05-4	644.11	$C_{12}H_6F_{21}O_4P$	b) 0.275	b) 0.55
	6:2 diPAP	6:2 polyfluoroalkyl phosphoric acid diesters	57677-95-9	790.17	$C_{16}H_9F_{26}O_4P$	a) 1.15<br>b) 0.31	a) 2.3<br>b) 0.62
	8:2 diPAP	8:2 polyfluoroalkyl phosphoric acid diesters	678-41-1	989.19	$C_{20}H_9F_{34}O_4P$	b) 0.12	b) 0.24
	6:2/8:2 diPAP	6:2/8:2 polyfluoroalkyl phosphoric acid diesters	943913-15-3	890.20	$C_{18}H_9F_{30}O_4P$	b) 0.70	b) 1.4
	10:2 diPAP	10:2 polyfluoroalkyl phosphoric acid diesters	1895-26-7	1190.23	$C_{24}H_9F_{42}O_4P$	b) 0.50	b) 1.0

Table S4 Internal Standards (IS) and Recovery Standards (RS) for a) 4000 QTRAP and b) TQ-S (and TQ-S µ for ADONA and GenX)

Standard	Abbreviation	Compound name	CAS number	Molar mass (g/mol)	Molecular formula
Internal Standard (IS)	IS-PFBA	perfluoro-n-[$^{13}C_4$]-butanoic acid	not available	218.01	$^{13}C_4HF_7O_2$
	IS-PFPeA	perfluoro-n-[3,4,5-$^{13}C_3$]-pentanoic acid	not available	267.03	$^{13}C_3{}^{12}C_2HF_9O_2$
	IS-PFHxA	perfluoro-n-[1,2-$^{13}C_2$]-hexanoic acid	not available	267.02	$^{13}C_2{}^{12}C_4HF_{11}O_2$
	IS-PFHpA	perfluoro-n-[1,2,3,4-$^{13}C_4$]-heptanoic acid	not available	368.03	$^{13}C_4{}^{12}C_3HF_{13}O_2$
	IS-PFOA	perfluoro-n-[1,2,3,4-$^{13}C_4$]-octanoic acid	not available	418.04	$^{13}C_4{}^{12}C_4HF_{15}O_2$
	IS-PFNA	perfluoro-n-[1,2,3,4,5-$^{13}C_5$]-nonanoic acid	not available	469.04	$^{13}C_5{}^{12}C_4HF_{17}O_2$
	IS-PFDA	perfluoro-n-[1,2-$^{13}C_2$]-decanoic acid	not available	516.07	$^{13}C_2{}^{12}C_8HF_{19}O_2$
	IS-PFUnDA	perfluoro-n-[1,2-$^{13}C_2$]-undecanoic acid	not available	566.07	$^{13}C_2{}^{12}C_9HF_{21}O_2$
	IS-PFDoDA	perfluoro-n-[1,2-$^{13}C_2$]-dodecanoic acid	not available	616.09	$^{13}C_2{}^{12}C_{10}HF_{23}O_2$
	IS-PFTeDA	perfluoro-n-[1,2-$^{13}C_2$]-tetradecanoic acid	not available	716.10	$^{13}C_2{}^{12}C_{12}HF_{27}O_2$
	IS-PFHxDA	perfluoro-n-[1,2-$^{13}C_2$]-hexadecanoic acid	not available	816.11	$^{13}C_2{}^{12}C_{14}HF_{31}O_2$
	IS-PFBS	Perfluoro-1-[2,3,4-$^{13}C_3$]-butane sulfonate	not available	302.07	$^{13}C_3{}^{12}CF_9SO_3$
	IS-PFHxS	perfluoro-n-hexane-[$^{18}O_2$]-sulfonate	not available	401.30	$C_6F_{13}S^{18}O_2{}^{16}O$
	IS-PFOS	perfluoro-n-[1,2,3,4-$^{13}C_4$]-octane sulfonate	not available	503.09	$^{13}C_4{}^{12}C_4F_{17}SO_3$
	IS- HFPO-DA	perfluoroether-[$^{13}C_3$]-carboxylic acid	not available	a) 333.03	a) $^{13}C_3{}^{12}C_3HF_{11}O_3$
	IS-PFOSA	perfluoro-n-[$^{13}C_8$]-octane sulfonamide	not available	507.09	$^{13}C_8H_2F_{17}NO_2S$

	IS-MeFOSA	N-methyl-d3-perfluoro-n-octane sulfonamide	not available	516.19	$C_9D_3HF_{17}NO_2S$
	IS-EtFOSE	N-deuterioethylperfluoro-n-octane sulfonamido-1,1,2,2-tetradeuterioethanol	not available	580.31	$C_{12}D_9HF_{17}NO_3S$
	IS-6:2 FTUCA	2H-perfluoro-[1,2-$^{13}C_2$]-2-octenoic acid	not available	360.07	$^{13}C_2{}^{12}C_6H_2F_{12}O_2$
	IS-8:2 FTUCA	2H-perfluoro-[1,2-$^{13}C_2$]-2-decenoic acid	not available	460.08	$^{13}C_2{}^{12}C_8H_2F_{16}O_2$
	IS-10:2 FTUCA	2H-perfluoro[1,2-$^{13}C_2$]-2-dodecenoic acid	not available	560.10	$^{13}C_2{}^{12}C_{10}H_2F_{20}O_2$
	IS-6:2 FTSA	1H,1H,2H,2H-perfluoro-[1,2-$^{13}C_2$]-octane sulfonate	not available	429.14	$^{13}C_2{}^{12}C_6H_4F_{13}SO_3$
	IS-8:2 FTSA	1H,1H,2H,2H-perfluoro-[1,2-$^{13}C_2$]-decane sulfonate	not available	529.13	$^{13}C_2{}^{12}C_8H_4F_{17}SO_3$
	IS-6:2 monoPAP	1H,1H,2H,2H-[1,2-$^{13}C_2$]-perfluoroalkyl phosphate	not available	444.05	$^{13}C_2{}^{12}C_6H_4F_{13}O_4P$
	IS-8:2 monoPAP	1H,1H,2H,2H-[1,2-13C2]-perfluorodecyl phosphate	not available	567.06	$^{13}C_2{}^{12}C_8H_4F_{17}O_4P$
	IS-6:2 diPAP	(bis-)1H,1H,2H,2H-[1,2-$^{13}C_2$]-perfluorooctyl-phosphate	not available	793.13	$^{13}C_4{}^{12}C_{12}H_8F_{26}O_4P$
	IS-8:2 diPAP	(bis-)1H,1H,2H,2H-[1,2-13C2]-perfluorodecyl-phosphate	not available	993.16	$^{13}C_4{}^{12}C_{16}H_8F_{34}O_4P$
	IS-EtFOSAA	N-ethyl-d5-perfluoro-1-octane sulfonamido acetic acid	not available	590.26	$C_{12}D_5H_3F_{17}NO_4S$

	RS-PFBA	perfluoro-n-[2,3,4-$^{13}C_3$]-butanoic acid	not available	217.02	$^{13}C_3{}^{12}CHF_7O_2$
	RS-PFPeA	perfluoro-n-[$^{13}C_5$]-pentanoic acid	not available	269.01	$^{13}C_5HF_9O_2$
Recovery Standard (RS)	RS-PFHxA	perfluoro-n-[1,2,3,4,6-$^{13}C_5$]-hexanoic acid	not available	319.02	$^{13}C_5{}^{12}C_1HF_{11}O_2$
	RS-PFOA	perfluoro-n-[$^{13}C_8$]-octanoic acid	not available	422.01	$^{13}O_8HF_{15}O_2$
	RS-PFNA	perfluoro-n-[$^{13}C_9$]-nonanoic acid	not available	473.01	$^{13}C_9HF_{17}O_2$

	RS-PFDA	perfluoro-n-[1,2,3,4,5,6-$^{13}C_6$]-decanoic acid	not available	520.04	$^{13}C_6{}^{12}C_4HF_{19}O_2$
	RS-PFUnDA	perfluoro-n-[1,2,3,4,5,6,7-$^{13}C_7$]-undecanoic acid	not available	571.04	$^{13}C_7{}^{12}C_4HF_{21}O_2$
	RS-PFHxS	perfluoro-n-[1,2,3-$^{13}C_3$]-hexane sulfonate	not available	402.08	$^{13}C_3{}^{12}C_3F_{13}SO_3$
	RS-PFOS	perfluoro-n-[$^{13}C_8$]-octane sulfonate	not available	507.06	$^{13}C_8F_{17}SO_3$
	RS-EtFOSA	N-ethyl-d5-perfluoro-1-octane sulfonamide	not available	532.23	$C_{10}D_5HF_{17}NO_2S$
	RS-MeFOSE	N-deuteriomethylperfluoro-1-octanesulfonamido-1,1,2,2,--tetradeuterioethanol	not available	564.27	$C_{11}{}^{2}H_7{}^{1}HF_{17}NO_3S$
	RS-4:2 FTSA	1H,1H,2H,2H-perfluoro-[1,2-$^{13}C_2$]-hexane sulfonate	not available	329.13	$^{13}C_2{}^{12}C_4H_4F_9SO_3$

Table S5 Blanks used for quality controls are reported for 500 µL MilliQ water for each extraction method and for both instruments a) 4000 QTRAP and b) TQ-S and TQ-S micro (ADONA and GenX)

Substance	blank SPE-WAX [ng/mL]	blank SPE-HLB [ng/mL]	blank ion-pair [ng/mL]
PFBA	b) <LOQ	a) <LOQ<br>b) <LOQ	b) <LOQ
PFPeA	b) <LOQ	a) <LOQ<br>b) <LOQ	b) <LOQ
PFHxA	b) <LOQ	a) <LOQ<br>b) <LOQ	b) <LOQ
PFHpA	b) <LOQ	a) <LOQ<br>b) <LOQ	b) <LOQ
PFOA	b) <LOQ	a) <LOQ<br>b) <LOQ	b) <LOQ
PFNA	b) <LOQ	a) <LOQ<br>b) <LOQ	b) <LOQ
PFDA	b) <LOQ	a) <LOQ<br>b) <LOQ	b) <LOQ
PFUnDA	b) <LOQ	a) <LOQ<br>b) <LOQ	b) <LOQ
PFDoDA	b) <LOQ	a) <LOQ<br>b) <LOQ	b) <LOQ
PFTrDA	b) <LOQ	a) <LOQ<br>b) <LOQ	b) <LOQ
PFTeDA	b) <LOQ	a) <LOQ<br>b) <LOQ	b) <LOQ
PFHxDA	b) <LOQ	b) <LOQ	b) <LOQ
PFOcDA	b) <LOQ	b) <LOQ	b) <LOQ

PFBS	b) <LOQ	a) <LOQ<br>b) <LOQ	b) <LOQ
PFPeS	b) <LOQ	a) <LOQ<br>b) <LOQ	b) <LOQ
PFHxS	b) <LOQ	a) <LOQ<br>b) <LOQ	b) <LOQ
PFHpS	b) <LOQ	a) <LOQ<br>b) <LOQ	b) <LOQ
PFOS	b) <LOQ	a) <LOQ<br>b) <LOQ	b) <LOQ
PFNS	b) <LOQ	a) <LOQ<br>b) <LOQ	b) <LOQ
PFDS	b) <LOQ	a) <LOQ<br>b) <LOQ	b) <LOQ
PFDoDS	b) <LOQ	b) <LOQ	b) <LOQ
PFHxPA	b) <LOQ	b) <LOQ	b) <LOQ
PFOPA	b) <LOQ	b) <LOQ	b) <LOQ
PFDPA	b) <LOQ	b) <LOQ	b) <LOQ
6:6 PFPiA	b) <LOQ	b) <LOQ	b) <LOQ
6:8 PFPiA	b) <LOQ	b) <LOQ	b) <LOQ
8:8 PFPiA	b) <LOQ	b) <LOQ	b) <LOQ
ADONA	b) <LOQ	a) <LOQ<br>b) <LOQ	b) <LOQ
HFPO-DA (GenX)	b) <LOQ	a) <LOQ<br>b) <LOQ	b) <LOQ

6:2 Cl-PFESA (major F-53B)	b) <LOQ	a) <LOQ<br>b) <LOQ	b) <LOQ
8:2 Cl-PFESA (minor F-53B)	b) <LOQ	b) <LOQ	b) <LOQ
PFECHS	b) <LOQ	b) <LOQ	b) <LOQ
FBSA	b) <LOQ	b) <LOQ	b) <LOQ
MeFBSA	b) <LOQ	b) <LOQ	b) <LOQ
PFOSA	b) <LOQ	a) <LOQ<br>b) <LOQ	b) <LOQ
MeFOSA	b) <LOQ	b) 0.0000	b) <LOQ
EtFOSA	b) <LOQ	a) <LOQ<br>b) <LOQ	b) <LOQ
MeFOSE	b) <LOQ	b) 0.0000	b) <LOQ
EtFOSE	b) <LOQ	a) <LOQ<br>b) <LOQ	b) <LOQ
MeFOSAA	b) <LOQ	b) 0.0000	b) <LOQ
EtFOSAA	b) <LOQ	a) <LOQ<br>b) <LOQ	b) <LOQ
FHxSA	b) <LOQ	b) 0.0000	b) <LOQ
MeFHxSA	b) <LOQ	b) 0.0000	b) <LOQ
3:3 FTCA	b) <LOQ	b) 0.0000	b) <LOQ
5:3 FTCA	b) <LOQ	b) 0.0000	b) <LOQ
7:3 FTCA	b) <LOQ	b) 0.0000	b) <LOQ

6:2 FTUCA	b) <LOQ	b) 0.0000	b) <LOQ
8:2 FTUCA	b) <LOQ	b) 0.0000	b) <LOQ
10:2 FTUCA	b) <LOQ	b) 0.0000	b) <LOQ
4:2 FTSA	b) <LOQ	a) <LOQ<br>b) <LOQ	b) <LOQ
6:2 FTSA	b) <LOQ	a) <LOQ<br>b) <LOQ	b) <LOQ
8:2 FTSA	b) <LOQ	a) <LOQ<br>b) <LOQ	b) <LOQ
10:2 FTSA	b) <LOQ	b) 0.0000	b) <LOQ
diSAmPAP	b) <LOQ	b) 0.0000	b) <LOQ
6:2 monoPAP	b) <LOQ	b) 0.0000	b) <LOQ
8:2 monoPAP	b) <LOQ	b) 0.0000	b) <LOQ
10:2 monoPAP	b) <LOQ	b) 0.0000	b) <LOQ
6:2 diPAP	b) <LOQ	a) <LOQ<br>b) <LOQ	b) <LOQ
8:2 diPAP	b) <LOQ	a) <LOQ<br>b) <LOQ	b) <LOQ
6:2/8:2 diPAP	b) <LOQ	a) <LOQ<br>b) <LOQ	b) <LOQ
10:2 diPAP	b) <LOQ	b) <LOQ	b) <LOQ

Table S6 The analytical parameters for the 4000 QTRAP, including the quantifier and qualifier ions for 31 native PFAS and for 14 isotope labelled PFAS, as well as the declustering potential (DP) and the collision energy (CE). The internal standards shown for each compound were used to correct for the recovery losses, and if no internal standard was available the mean recovery of three native PFAS was used to correct for the recovery losses instead

**Compound**	**Precursor/ Product Ions Quantification (m/z)**	**DP (V)**	**CE (eV)**	**Precursor/ Product Ions Qualification (m/z)**	**DP (V)**	**CE (eV)**	**Internal Standard**
PFBA	213/169	-30	-12	213/147	-30	-10	$^{13}C_4$-PFBA
PFPeA	263/219	-30	-12	263/197	-30	-10	($^{13}C_4$-PFBA * $^{13}C_2$-PFHxA)/2
PFHxA	313/269	-35	-14	313/119	-35	-28	$^{13}C_2$-PFHxA
PFHpA	363/319	-30	-14	363/169	-30	-24	($^{13}C_2$-PFHxA * $^{13}C_4$-PFOA)/2
PFOA	413/369	-65	-14	413/169	-65	-26	$^{13}C_4$-PFOA
PFNA	463/419	-50	-14	463/219	-50	-22	$^{13}C_5$-PFNA
PFDA	513/469	-45	-16	513/219	-45	-26	$^{13}C_2$-PFDA
PFUnDA	563/519	-60	-16	563/269	-60	-24	$^{13}C_2$-PFUnDA
PFDoDA	613/569	-45	-14	613/169	-45	-38	$^{13}C_2$-PFDoDA
PFTrDA	663/619	-50	-18	663/169	-50	-38	mean PFTrDA recovery from three QC
PFTeDA	713/669	-70	-24	713/169	-70	-42	mean PFTeDA recovery from three QC
PFBS	299/80	-55	-46	299/99	-55	-46	$^{18}O_2$-PFHxS
PFPeS	349/80	-85	-62	349/99	-85	-44	$^{18}O_2$-PFHxS
PFHxS	399/80	-40	-66	399/99	-40	-66	$^{18}O_2$-PFHxS
PFHpS	449/80	-40	-66	449/99	-40	-66	($^{18}O_2$-PFHxS * $^{13}C_4$-PFOS)/2
PFOS	499/80	-65	-110	499/99	-65	-84	$^{13}C_4$-PFOS
PFNS	549/80	-140	-100	549/99	-140	-78	$^{13}C_4$-PFOS

PFDS	599/80	-105	-112	599/99	-105	-94	$^{13}C_4$-PFOS
FOSA	498/78	-80	-82	498/64	-80	-128	^{2}d-EtFOSAA
EtFOSA	526/169	-75	-40	526/219	-75	-36	^{2}d-EtFOSA
EtFOSAA	584/419	-90	-28	584/526	-90	-28	^{2}d-EtFOSAA
EtFOSE	630/59	-50	-62				^{2}d-EtFOSE
4:2 FTSA	327/307	-115	-28	327/81	-115	-52	$^{13}C_2$-6:2 FTSA
6:2 FTSA	427/81	-160	-64	427/407	-160	-32	$^{13}C_2$-6:2 FTSA
8:2 FTSA	527/81	-145	-76	527/507	-145	-38	$^{13}C_2$-6:2 FTSA
6:2 Cl-PFESA	531/351	-105	-38	531/35	-105	-82	$^{13}C_4$-PFOS
6:2 diPAP	789/97	-120	-76	789/79	-120	-128	mean 6:2 diPAP recovery from three QC
6:2/8:2 diPAP	889/97	-145	-76	889/79	-145	-130	$^{13}C_4$-8:2 diPAP
8:2 diPAP	989/97	-140	-84	989/79	-140	-126	$^{13}C_4$-8:2 diPAP
HFPO-DA (GenX)	329/285	-45	-8	329/169	-45	-18	mean GenX recovery from three QC
ADONA	377/251	-70	-18	377/85	-70	-54	$^{13}C_4$-PFOA
$^{13}C_4$-PFBA	217/172	-30	-12				
$^{13}C_2$-PFHxA	315/270	-35	-14				
$^{13}C_4$-PFOA	417/372	-65	-14				
$^{13}C_5$-PFNA	469/423	-50	-14				
$^{13}C_2$-PFDA	515/470	-45	-16				
$^{13}C_2$-PFUnDA	565/520	-60	-16				
$^{13}C_2$-PFDoDA	615/570	-45	-14				
$^{18}O_2$-PFHxS	403/84	-40	-66	403/103	-40	-66	
$^{13}C_4$-PFOS	503/80	-65	-110	503/99	-65	-84	
^{2}H -EtFOSA	531/169	-75	-40				

^{2}H -EtFOSAA	589/419	-100	-28	589/531	-100	-28
^{2}H -EtFOSE	630/59	-50	-62	630/606	-50	-60
$^{13}C_{2}$-6:2 FTSA	429/81	-160	-64	429/80	-160	-82
$^{13}C_{4}$-8:2 diPAP	993/97	-140	-84	993/79	-140	-126

Table S7 The analytical parameters for the TQ-S and TQ-S micro, including the quantifier and qualifier ions for 61 native PFAS and 37 isotope labeled PFAS (28 internal standards and 9 recovery standards), as well as the cone voltage (CV) and the collision energy (CE). The internal standards shown for each compound were used to correct for the recovery losses, and if no internal standard was available the mean recovery of three native PFAS was used to correct for the recovery losses instead. Elements for which no specific parameter was available were left blank

Compound	Precursor/ Product Ions Quantification (m/z)	CV (V)	CE (eV)	Precursor/ Product Ions Qualification (m/z)	CV (V)	CE (eV)	Internal Standard
PFBA	213/169	20	11				$^{13}C_4$-PFBA
PFPeA	263/219	20	8				$^{13}C_3$-PFPeA
PFHxA	313/269	20	9	313/119	20	26	$^{13}C_2$-PFHxA
PFHpA	363/319	20	10	363/169	20	16	$^{13}C_4$-PFHpA
PFOA	413/369	20	10	413/169	20	18	$^{13}C_4$-PFOA
PFNA	463/419	20	12	463/219	20	18	$^{13}C_5$-PFNA
PFDA	513/469	20	11	513/219	20	18	$^{13}C_2$-PFDA
PFUnDA	563/519	20	12	563/269	20	18	$^{13}C_2$-PFUnDA
PFDoDA	613/569	34	14	613/169	40	22	$^{13}C_2$-PFDoDA
PFTrDA	663/619	20	14	663/169	20	26	mean PFTrDA recovery from three QC
PFTeDA	713/669	20	14	713/169	20	28	$^{13}C_2$-PFTeDA
PFHxDA	813/769	30	15	813/169	42	32	$^{13}C_2$-PFHxDA
PFOcDA	913/869	36	15	913/169	36	36	mean PFOcDA recovery from three QC
PFBS	299/99	20	26	299/80	20	26	$^{13}C_3$-PFBS
PFPeS	349/99	20	26	349/80	20	30	(^{13}C-PFBS * ^{18}O-PFHxS)/2
PFHxS	399/99	20	30	399/80	20	34	$^{18}O_2$-PFHxS
PFHpS	449/99	20	30	449/80	20	35	(^{18}O-PFHxS *

							^{13}C-PFOS)/2
PFOS	499/99	20	38	499/80, 499/169	20	44, 34	$^{13}C_4$-PFOS
PFNS	549/99	20	38	549/80	20	44	^{13}C-PFOS
PFDS	599/99	20	42	599/80	20	58	^{13}C-PFOS
PFDoDS	699/99	20	40	699/80	20	45	mean PFDoDS recovery from three QC
FOSA	498/78	82	30	498/169	82	29	$^{13}C_8$-FOSA
MeFOSA	512/169	27	45				^{3}d-MeFOSA
EtFOSA	526/169	27	45				mean EtFOSA recovery from three QC
MeFOSAA	570/419	16	18	570/512	18	22	mean FOSAA recovery from three QC
EtFOSAA	584/419	18	20	584/483	18	16	mean EtFOSAA recovery from three QC
MeFOSE	556/122	42	34	616/59	27	45	mean MeFOSE recovery from three QC
EtFOSE	570/136	48	32	630/59	27	45	^{9}d-EtFOSE
3:3 FTCA	241/176	10	8	241/117	10	30	mean 3:3 FTCA recovery from three QC
5:3 FTCA	341/237	10	16	341/217	10	22	mean 5:3 FTCA recovery from three QC
7:3 FTCA	441/337	12	14	441/317	12	20	mean 7:3 FTCA recovery from three QC
6:2 FTUCA	357/293	10	18	357/243	10	36	$^{13}C_2$-6:2 FTUCA
8:2 FTUCA	457/393	10	18	457/393	10	38	$^{13}C_2$-8:2 FTUCA
10:2 FTUCA	557/493	8	16	557/243	8	38	$^{13}C_2$-10:2 FTUCA
FBSA	298/78	20	20	298/119	20	15	mean FBSA recovery from three QC

MeFBSA	312/112	14	20	312/219	14	16	mean MeFBSA recovery from three QC
FHxSA	398/78	30	26	398/169	30	28	mean PFHxSA recovery from three QC
MeFHxSA	412/169	24	24	412/319	24	20	mean MeFHxSA recovery from three QC
4:2 FTSA	327/307	20	20	327/81	20	28	$^{13}C_2$-6:2 FTSA
6:2 FTSA	427/407	20	20	427/81	20	28	$^{13}C_2$-6:2 FTSA
8:2 FTSA	527/507	20	20	527/80	20	28	$^{13}C_2$-8:2 FTSA
10:2 FTSA	627/607	20	20	627/80	20	28	mean 10:2 FTSA recovery from three QC
6:2 Cl-PFESA	531/351	58	24	531/83	58	24	mean 6:2 Cl-PFESA recovery from three QC
8:2 Cl-PFESA	631/451	58	24	631/83	58	24	mean 8:2 Cl-PFESA recovery from three QC
PFECHS	461/381	2	24	461/99	2	26	mean PFECHS recovery from three QC
6:2 mPAP	443/97	10	18	443/79	10	38	$^{13}C_2$-6:2mPAP
8:2 mPAP	543/97	22	14	543/79	22	56	$^{13}C_2$-8:2 mPAP
10:2 mPAP	643/97	24	22	643/79	24	28	mean 10:2 mPAP recovery from three QC
6:2 diPAP	789/97	64	28	789/443	64	18	^{13}C-6:2 diPAP
6:2/8:2 diPAP	889/97	66	34	889/443, 889/543	66	26	mean 6:2/8:2 diPAP recovery from three QC
8:2 diPAP	989/97	68	34	989/543	68	26	^{13}C-8:2 diPAP
10:2 diPAP	1189/97	68	34	1189/643	68	26	mean 10:2 diPAP recovery from three QC
SAmPAP	650/526	2	24	650/97	2	26	mean SAmPAP recovery from three QC

diSAmPAP	1203/526	92	46	1203/169	92	64	mean diSAmPAP recovery from three QC
PFHxPA	3997/79	62	26				mean PFHxPA recovery from three QC
PFOPA	499/79	62	30				mean PFOPA recovery from three QC
PFDPA	599/79	62	30				mean PFDPA recovery from three QC
C6/C6 PFPiA	701/401	62	28				mean C6/C6 PFPiA recovery from three QC
C6/C8 PFPiA	801/401	24	28	801/501	24	28	mean C6/C8 PFPiA recovery from three QC
C8/C8 PFPiA	901/501	24	28	901/601, 901/401	24	28	mean C8/C8 PFPiA recovery from three QC
HFPO-DA (GenX)	285/169	20	7	329/285	15	5	$^{13}C_3$-HFPO-DA
ADONA	377/251	15	13	377/85	15	29	^{13}C-PFOA
$^{13}C_4$-PFBA (IS)	217/172	20	11				
$^{13}C_3$-PFBA (RS)	216/172	20	11				
$^{13}C_3$-PFPeA (IS)	266/222	20	8				
$^{13}C_5$-PFPeA (RS)	268/223	20	8				
$^{13}C_2$-PFHxA (IS)	315/270	20	9				
$^{13}C_5$-PFHxA (RS)	318/273	20	9				
$^{13}C_4$-PFHpA (IS)	367/322	20	10				
$^{13}C_4$-PFOA (IS)	417/372	20	10				
$^{13}C_8$-PFOA (RS)	421/376	20	10				
$^{13}C_5$-PFNA (IS)	468/423	20	12				

$^{13}C_9$-PFNA (RS)	472/427	19	12
$^{13}C_2$-PFDA (IS)	515/470	20	11
$^{13}C_6$-PFDA (RS)	519/474	20	11
$^{13}C_2$-PFUnDA (IS)	565/520	20	12
$^{13}C_7$-PFUnDA (RS)	570/525	20	12
$^{13}C_2$-PFDoDA (IS)	615/570	34	14
$^{13}C_2$-PFTeDA (IS)	715/670	20	14
$^{13}C_2$-PFHxDA (IS)	815/770	30	15
$^{13}C_3$-PFBS (IS)	302/99	20	26
$^{18}O_2$-PFHxS (IS)	403/103	20	30
$^{13}C_3$-PFHxS (RS)	402/99	20	30
$^{13}C_4$-PFOS (IS)	503/99	20	38
$^{13}C_8$-PFOS (RS)	507/99	20	38
$^{13}C_8$-FOSA (IS)	506/78	82	30
^{3}d-MeFOSA (IS)	515/169	27	45
^{5}H-EtFOSA (RS)	531/169	27	45
^{5}d-Et-FOSAA (IS)	589/419	18	20
^{7}d-MeFOSE (RS)	623/59	27	45
^{9}d-EtFOSE (IS)	639/59	27	45
$^{13}C_2$-6:2 FTUCA (IS)	359/294	10	18
$^{13}C_2$-8:2 FTUCA (IS)	459/343	10	38
$^{13}C_2$-10:2 FTUCA (IS)	559/494	8	16

$^{13}C_2$-4:2 FTSA (RS)	329/81	20	28			
$^{13}C_2$-6:2 FTSA (IS)	429/409	20	20			
$^{13}C_2$-8:2 FTSA (IS)	529/509	20	20			
$^{13}C_3$-HFPO-DA (IS)	287/167	20	7			
$^{13}C_2$-6:2mPAP (IS)	445/79	10	38	445/97	10	18
$^{13}C_2$-8:2 mPAP (IS)	545/79	22	56	545/97	22	14
^{13}C-6:2 diPAP (IS)	789/543	64	18	789/643	64	18
^{13}C-8:2 diPAP (IS)	989/643	68	26	989/743	68	26

Table S8 Descriptive statistics for maternal serum samples (n=8)

Parameters	Substances and concentrations in µg/L								
	PFOA	PFNA	PFDA	PFUnDA	PFDoDA[§]	PFBS	PFHxS	PFHpS	PFOS
min.	≥ LOD	0.076	0.062	< LOD	< LOD	< LOD	0.13	< LOD	0.27
median	0.46	0.19	0.089	0.049	0.0000	0.0000	0.23	0.0000	0.37
mean	0.69	0.19	0.10	0.051	0.061	0.0018	0.23	0.0079	0.60
95th percentile	2.1	0.30	0.17	0.11	0.21	0.010	0.36	0.035	1.2
max.	2.9	0.33	0.20	0.13	0.26	0.014	0.37	0.042	1.2

[§] for PFDoDA values from 4000 QTRAP

Statistically significant correlations (Spearman´s correlation coefficients, r) between the concentrations of PFOA and other long-chain PFCAs in the maternal serum samples were identified: between PFOA and PFNA (r=0.90), PFOA and PFDA (r=0.83), and PFOA and PFUnDA (r=0.73). Additionally, a statistically significant correlation between PFOS and PFHpS levels (r=0.76) were identified in maternal serum samples. Comparing the results of the maternal and cord serum samples, PFOA correlated significantly positive correlations for PFOA (r=0.90) were found. Statistically significant correlations were also identified for PFDA (r=0.84). Pearson´s correlation coefficients with significant positive correlations were found for PFNA and PFDA (0.90), and for PFDA and PFUnDA (0.89). PFOS significantly correlated between maternal and cord serum samples with a Pearson´s correlation coefficients of 0.76. These significant positive associations can indicate a common exposure pathway [5]. No correlations were observed for any of the PFAAs with PFDoDA and between PFCAs and PFSAs in the maternal serum samples. It has to be noted that because of the small sample sizes the interpretation of the results have to be conducted with care.

For the evaluation of inorganic fluorine elimination, 100 ng inorganic fluorine (IF) were measured in 1 mL methanol (IF QC) with CIC; 100 ng IF were spiked into 3 g human placental tissue (n=2) for testing the placenta II method; 100 ng IF were spiked into 2 mL bovine serum (n=2) for testing the SPE-WAX method; and 100 ng IF were spiked into 1 mL MilliQ (n=1) for testing the ion-pair method. For the placenta II and SPE-WAX methods, the same placental tissue and bovine serum without spiking IF were also extracted and analyzed with CIC. Results suggested that no IF were co-extracted from the tested methods (Table S9).

Table S9 Evaluation of inorganic fluorine elimination of different extraction methods with different matrices: 100 ng inorganic fluorine (IF) were measured in 1 mL methanol (IF QC), 100 ng IF were spiked into 3 g human placental tissue (n=2) for placenta II method, 100 ng IF were spiked into 2 mL bovine serum (n=2) for SPE-WAX method, and 100 ng F into 1 mL of Milli-Q water for the ion pair method. Values shown in the table are the average two measurements for the placenta II and SPE-WAX methods

Method	Concentrations in ng F/ mL				Reference
	Matrix	IF QC	Spiked with IF	Without IF	
Placenta II	placenta	98.1	9.5	11.4	Miaz and co-workers [6]
SPE-WAX	bovine serum	102.3	58.3	54.5	Miyake and co-worker [3]
SPE-HLB			not available	not available	not available
LLE (“ion pair”)	Milli-Q water	95	<LOD	<LOD	Miyake and co-worker [7]

Abbreviations

6:6 PFPiA	bis-perfluorohexylphosphinate
6:8 PFPiA	perfluorohexyl-perfluorooctyl phosphinate
8:2 Cl-PFESA	6:2 chlorinated polyfluorinated ether sulfonate (major F-53B)
8:2 Cl-PFESA	8:2 chlorinated polyfluorinated ether sulfonate (minor F-53B)
8:8 PFPiA	bis-perfluorooctyl phosphinate
ADONA	dodecalfluoro-3H-4,8-dioxanonanoate
CIC	combustion ion chromatography
diPAP	polyfluoroalkyl phosphate diester
diSAmPAP	(bis-)2-N-ethylperfluorooctane-1-sulfonamido-ethyl-phosphate
EOF	extractable organic fluorine
ESI	electrospray ionization
EtFOSA	N-ethyl-perfluoro-1-octane sulfonamide
EtFOSAA	N-ethyl-perfluoro-1-octane sulfonamido acetic acid
EtFOSE	N-ethyl-perfluoro-1-octane sulfonamido ethanol
FBSA	perfluorobutane sulfonamide
FOSA	perfluoro-1-octane sulfonamide
FOSAs	perfluorooctane sulfonamides
FOSEs	perfluorooctane sulfonamidoethanols
FTCA	fluorotelomer carboxylate
FTOHs	fluorotelomer alcohols
FTSA	fluorotelomer sulfonate
FTUCA	fluorotelomer unsaturated carboxylate

fTW	filtered-tap-water
HF	hydrogen fluoride
HFPO-DA	perfluoroether carboxylic acid (GenX)
HPLC-MS/MS	high-performance liquid chromatography tandem-mass spectrometry
LOD	limit of detection
LOQ	limit of quantification
MeFBSA	N-methyl-perfluorobutane sulfonamide
MeFOSA	N-methyl-perfluoro-1-octne sulfonamide
MeFOSAA	N-methyl-perfluoro-1-octane sulfonamido acetic acid
MeFOSE	N-methyl-perfluoro-1-octane sulfonamido ethanol
MePFHxSA	N-methyl-perfluoro-1-hexane sulfonamide
monoPAP	polyfluoroalkyl phosphate monoester
PAPs	polyfluoroalkyl phosphate esters
PASF	perfluoroalkyl sulfonyl fluoride
PFAAs	perfluoroalkyl acids
PFBA	perfluoro-n-butanoate (perfluoro-n-butanoic acid)
PFBS	perfluoro-1-butane sulfonate
PFCA	perfluoroalkyl carboxylic acid (perfluoroalkyl carboxylate)
PFDA	perfluoro-n-decanoate (perfluoro-n-decanoic acid)
PFDoDA	perfluoro-n-dodecane carboxylate (perfluoro-n-decanoic acid)
PFDoDS	perfluoro-1-dodecansulfonate
PFDPA	perfluorodecyl phosphonate
PFDS	perfluoro-1-decane sulfonate
PFECA	perfluoroether carboxylate (carboxylic acid)

305	PFECHS	perfluoro-4-ethylcyclohexane sulfonate
306	PFESA	perfluoroether sulfonate (perfluoroether sulfonic acid)
307	PFHpA	perfluoro-n-heptane carboxylate (perfluoro-n-heptanoic acid)
308	PFHpS	perfluoro-1-heptane sulfonate
309	PFHxA	perfluoro-n-hexane carboxylate (perfluoro-n-hexanoic acid)
310	PFHxDA	perfluoro-n-hexadecanoate
311	PFHxPA	perfluorohexylphosphinate
312	PFHxS	perfluoro-1-hexane sulfonate
313	PFHxSA	perfluoro-1-hexanesulfonamide
314	PFNA	perfluoro-n-nonane carboxylate (perfluoro-n-nonanoic acid)
315	PFNS	perfluoro-1-nonane sulfonate
316	PFOA	perfluorooctanoate (perfluoro-n-octanoic acid)
317	PFOcDA	perfluoro-1-octadecanoic acid
318	PFOPA	perfluorooctylphosphonate
319	PFOS	perfluorooctane sulfonate
320	PFPA	perfluorophosphonates
321	PFPeA	perfluoro-n-pentane carboxylate (perfluoro-n-pentanoic acid)
322	PFPeS	perfluoro-1-pentane sulfonate
323	PFPiA	perfluorophosphinates
324	PFSA	perfluoroalkane sulfonic acid (perfluoroalkane sulfonate)
325	PFTeDA	perfluoro-n-tetradecane carboxylate (perfluoro-n-tetranoic acid)
326	PFTrDA	perfluoro-n-tridecane carboxylate (perfluoro-n-tridecanoic acid)
327	PFUnDA	perfluoro-n-undecane carboxylate (perfluoro-n-undecanoic acid)
328	POPs	persistent organic pollutants

SPE-HLB	solid phase extraction with hydrophilic lipophilic balance
SPE-WAX	solid phase extraction with weak anion exchange
UPLC	ultra-performance liquid chromatography

References

1. Kuklenyik Z, Reich JA, Tully JS, et al (2004) Automated Solid-Phase Extraction and Measurement of Perfluorinated Organic Acids and Amides in Human Serum and Milk. Environ Sci Technol 38:3698–3704

2. Benskin JP, Bataineh M, Martin JW (2007) Simultaneous Characterization of Perfluoroalkyl Carboxylate, Sulfonate, and Sulfonamide Isomers by Liquid Chromatography-Tandem Mass Spectrometry. Anal. Chem. 79:6455–6464. https://doi.org/10.1021/ac070802d

3. Miyake Y, Yamashita N, So MK, et al (2007) Determination of trace levels of total fluorine in water using combustion ion chromatography for fluorine: A mass balance approach to determine individual perfluorinated chemicals in water. J. Chromatogr. A 1143 (1-2):98–104

4. Hansen KJ, Clemen LA, Ellefson M, Johnson HO (2001) Compound-Specific, Quantitative Characterization of Organic Fluorochemicals in Biological Matrices. Environ. Sci. Technol. 35 (4):766–770

5. Wu M, Sun R, Wang M, et al (2017) Analysis of perfluorinated compounds in human serum from the general population in Shanghai by liquid chromatography-tandem mass spectrometry (LC-MS/MS). Chemosphere 168:100–105

6. Miaz LT, Plassmann MM, Gyllenhammar I, et al (2020) Temporal trends of suspect- and target-per/polyfluoroalkyl substances (PFAS), extractable organic fluorine (EOF) and total fluorine (TF) in pooled serum from first-time mothers in Uppsala, Sweden, 1996–2017. Environ Sci Process Impacts 22:1071–1083. https://doi.org/10.1039/C9EM00502A

7. Miyake Y, Yamashita N, Rostkowski P, et al (2007) Trace analysis of total fluorine in human blood using combustion ion chromatography for fluorine: A mass balance approach for the determination of known and unknown organofluorine compounds. J. Chromatogr. A 1154:214–221
